# OCT4-induced oligodendrocyte progenitor cells promote remyelination and ameliorate disease

**DOI:** 10.1038/s41536-021-00199-z

**Published:** 2022-01-13

**Authors:** Wonjin Yun, Kyung-Ah Choi, Insik Hwang, Jie Zheng, Minji Park, Wonjun Hong, Ah-Young Jang, Jeong Hee Kim, Wonji Choi, Dae-Sung Kim, In Yong Kim, Yong Jun Kim, Ying Liu, Byung Sun Yoon, Gyuman Park, Gwonhwa Song, Sunghoi Hong, Seungkwon You

**Affiliations:** 1 Department of Biotechnology, College of Life Sciences and Biotechnology, Korea University, Seoul, 136-701 Republic of Korea; 2 Institute of Animal Molecular Biotechnology, Korea University, Seoul, 136-701 Republic of Korea; 3 School of Biosystem and Biomedical Science, College of Health Science, Korea University, Seoul, Republic of Korea; 4 Department of Biomedical Science, Graduate School, Kyung Hee University, Seoul, Republic of Korea; 5 Laboratory of Reprogramming & Differentiation, Department of Biotechnology, College of Life Science and Biotechnology, Korea University, Seoul, 136-701 Republic of Korea; 6 Department of Pathology, College of Medicine, Kyung Hee University, Seoul, Republic of Korea; 7 Department of Neurosurgery, the University of Texas Health Science Center at Houston, Houston, TX USA; 8 Institute of Regenerative Medicine, STEMLAB, Inc., Seoul, 02841 Republic of Korea; 9 Department of Integrated Biomedical and Life Science, College of Health Science, Korea University, Seoul, Republic of Korea

**Keywords:** Reprogramming, Regeneration and repair in the nervous system

## Abstract

The generation of human oligodendrocyte progenitor cells (OPCs) may be therapeutically valuable for human demyelinating diseases such as multiple sclerosis. Here, we report the direct reprogramming of human somatic cells into expandable induced OPCs (iOPCs) using a combination of OCT4 and a small molecule cocktail. This method enables generation of A2B5^+^ (an early marker for OPCs) iOPCs within 2 weeks retaining the ability to differentiate into MBP-positive mature oligodendrocytes. RNA-seq analysis revealed that the transcriptome of O4^+^ iOPCs was similar to that of O4^+^ OPCs and ChIP-seq analysis revealed that putative OCT4-binding regions were detected in the regulatory elements of CNS development-related genes. Notably, engrafted iOPCs remyelinated the brains of adult shiverer mice and experimental autoimmune encephalomyelitis mice with MOG-induced 14 weeks after transplantation. In conclusion, our study may contribute to the development of therapeutic approaches for neurological disorders, as well as facilitate the understanding of the molecular mechanisms underlying glial development.

## Introduction

The mammalian central nervous system (CNS) consists of a diverse array of cell types, including neurons, astrocytes, and oligodendrocytes, which mutually interact within the sophisticated structure and dynamics of neural networks. Oligodendrocytes ensure axonal stability by generating myelin sheaths around the axons of most neurons and induce the local accumulation of neurofilaments, allowing the faster electrical conduction of impulses^1^. Chronic demyelination in the CNS leads to irreversible axonal damage and is a major cause of disability from diseases such as multiple sclerosis, multiple system atrophy, and inherited leukodystrophies^2,3^. The transplantation of oligodendrocyte progenitor cells (OPCs), which are committed to the oligodendrocyte lineage and capable of participating in the myelin repair process, has been considered a promising therapeutic approach for CNS demyelinating diseases^4^. However, due to the poor accessibility and availability of natural glial cell sources, current therapeutic attempts are limited to the transplantation of autologous mesenchymal stem cells or the administration of several therapeutic agents; these treatments have only limited therapeutic success^5^. During the last decade, substantial advances have been achieved in differentiating human pluripotent stem cells, including human embryonic stem cells (hESCs)^6–8^ and induced pluripotent stem cells (iPSCs)^9–11^ into OPCs. Notwithstanding their great achievements in the establishment of rapid, efficient, and highly reproducible protocol^12^, these approaches require a long-term differentiation over three months. In this regard, several studies have been published on lineage-specific factor-driven generation of induced OPCs (iOPCs) from rodent fibroblasts^13,14^ and human neural stem cells (NSCs)^15,16^ within a period of three weeks. Among the used factors, a combination of SOX10, OLIG2, and NKX6.2 (SON) was commonly used in both rodent and human^13,15^, but the feasibility on applying these methods to human somatic cells remains uncertain.

In this study, we identified the role of OCT4, a master regulator of pluripotency, in combination with small molecules, serving as the critical cue governing the reprogramming of human somatic cells into OPCs, using RNA-seq and ChIP-seq. The generated iOPCs promoted remyelination in the hypomyelinated shiverer mice and rescued the disease phenotype in a mouse model of multiple sclerosis (MS). Thus, our approach for generating iOPCs may be a resource for establishing therapeutic transplantation as a treatment for various demyelinating diseases and provide new insights into underlying glial development.

## Results

### Screening of chemical compounds for generation of iOPCs

Previously, we have developed the culture condition “ATPV” containing A83-01, thiazovivin, purmorphamine, and valproic acid (VPA) which could generate chemically induced neural stem cells (ciNSCs) from mouse embryonic fibroblasts^17^. Based on our previous findings, we investigated whether the chemical cocktail “ATPV” can elicit reprogramming of human fibroblasts (BJ) and SOX10::eGFP fibroblasts^18^ into OPCs by forced expression of SON with additional transcriptional factors (NKX2.2, ID2, and ID4). However, this strategy was not sufficient for successful generation of OPCs from human fibroblasts in the designed conditions (Supplementary Fig. 1a, b). To facilitate the reprogramming process, we employed transcription factors that have the strongest effect on direct reprogramming^19–22^. Surprisingly, we observed small round cells with a bipolar morphology consistent with OPCs in BJ and expression of GFP in SOX10::eGFP fibroblasts at 7 days post-transduction of OCT4 (Supplementary Fig. 1c). These cells exhibited increased expression of OLIG2 and SOX10, regarded as a strong indicator of successful conversion into OPCs^13–15^ (Supplementary Fig. 2a) and also expressed A2B5, a typical surface marker of OPC (Supplementary Fig. 2b). Based on these results, we hypothesized that human OPCs can be directly generated from fibroblasts in the presence of a combination of OCT4 and small molecules.

**Fig. 1:**
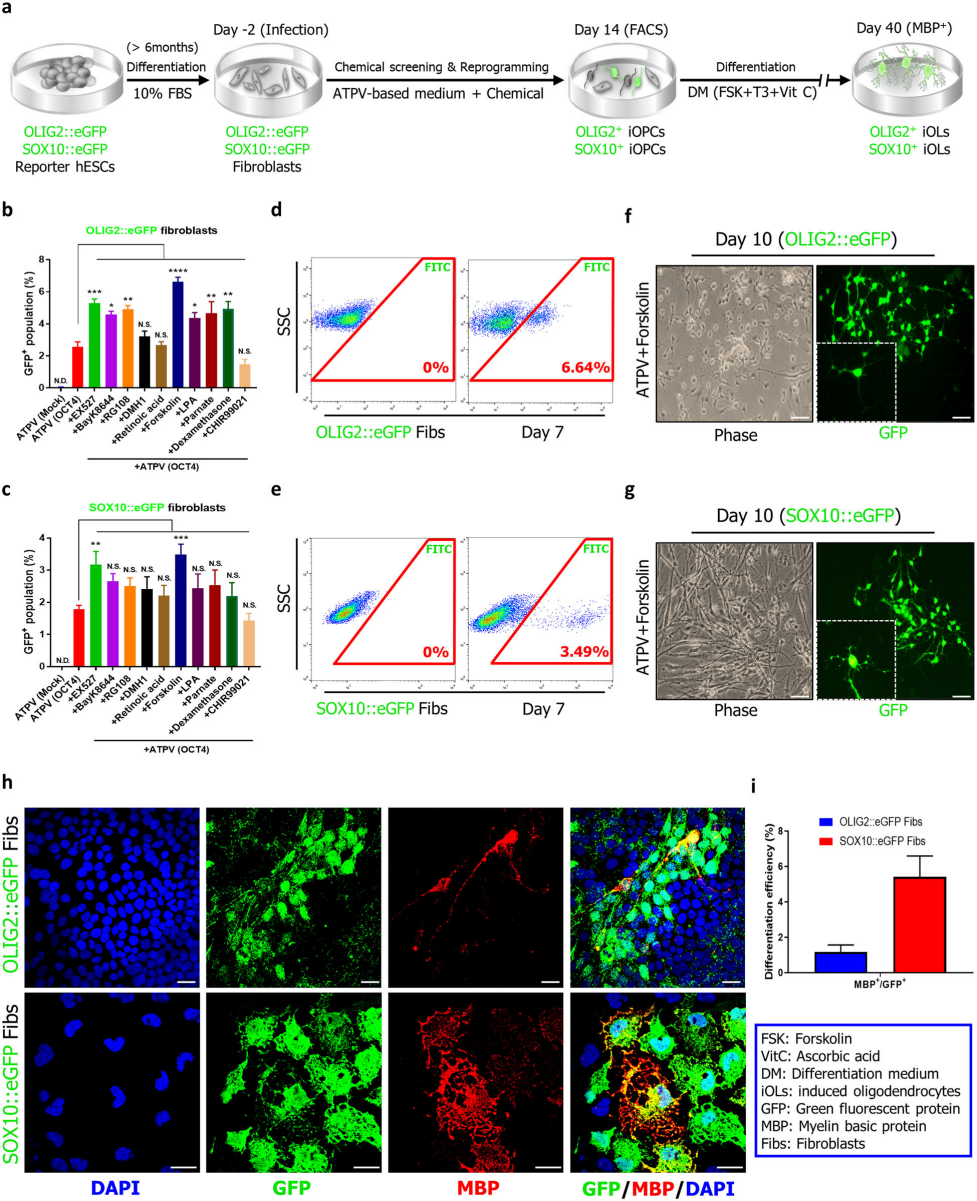
Screening for inducing OLIG2^+^ and SOX10^+^ oligodendrocyte progenitor cells from human fibroblasts. **a** A schematic representation of the protocol followed for the direct conversion of hESC-derived fibroblasts to OLIG2^+^ and SOX10^+^ oligodendrocyte progenitor cells. The OLIG2 and SOX10::eGFP fibroblasts were transduced with OCT4 and cultured in ATPV-based medium containing each of additional small molecules. To differentiate into oligodendrocytes, the medium was changed to differentiation medium (DM) containing forskolin (FSK), T3, and ascorbic acid (Vit C). **b**, **c** Quantitation of GFP^+^ populations in flow cytometry analysis following the induction of OLIG2::eGFP and SOX10::eGFP fibroblasts with OCT4 under various chemical combinations in culture. The data from four independent experiments are presented as the mean + SD. *, statistically significant difference vs. the ATPV culture condition. Significant differences were analyzed by one-way ANOVA. N.S., not significant; N.D., not detected. *P < 0.05, **P < 0.01, ***P < 0.001, ****P < 0.0001. **d**, **e** Representative flow cytometry data for GFP^+^ cells in OCT4-induced OLIG2::eGFP and SOX10::eGFP fibroblasts cultured in the presence of ATPV and forskolin. The data from three independent experiments are presented as the mean values. **f**, **g** Representative phase contrast (left), and green fluorescence intensity images (right) for GFP^+^ cells in OCT4-induced OLIG2::eGFP and SOX10::eGFP fibroblasts cultured in the presence of ATPV and forskolin. Scale bars, 100 µm. The boxed area in the image on the left represents a magnified (3×) picture of GFP^+^ cells. **h** Representative fluorescence images for the differentiation of OCT4-induced OLIG2::eGFP and SOX10::eGFP fibroblasts cultured in the presence of ATPV and forskolin into oligodendrocytes on day 60. The GFP^+^ cells coexpressed MBP. Scale bars, 100 µm. **i** Quantification of MBP-positive cells in GFP-positive cells on day 60.

To further optimize the reprogramming process, we screened additional small molecules, including modulators of epigenetic enzymes and signaling pathways involved in oligodendrocyte differentiation (Supplementary Table 1), using hESC-OLIG2::eGFP and hESC-SOX10::eGFP reporter systems^18,23^. We transduced OCT4 into hESC-derived OLIG2::eGFP and SOX10::eGFP fibroblasts that lacked neural or pluripotency markers (Supplementary Fig. 3a) using a protocol outlined in Fig. 1a and determined if additional small molecules could augment ATPV-enabled reprogramming based on GFP expression. As anticipated, GFP expression was detected in OCT4-transduced ATPV cultures on day 7 after transduction but not detected in cultures infected with control viruses (Fig. 1b, c). Notably, we found that the addition of forskolin, which is known to modulate the differentiation of NSCs into OPCs^24^, significantly increased GFP^+^ populations (Fig. 1b−g); the removal of each of components including small molecules and growth factors from forskolin-containing medium decreased reprogramming efficiency (Supplementary Fig. 3b−e). In addition, isolated GFP^+^ cells in reporter cell lines had typical bipolar morphology (Supplementary Fig. 4a, b) and expressed several OPC markers (Supplementary Fig. 4c). When cultured in differentiation medium, MBP^+^ cells could be detectable on day 40 (day of the overall culture) and 1.18 ± 0.41% GFP^+^ cells in OLIG2::eGFP fibroblasts and 5.43 ± 1.18% GFP^+^ cells in SOX10::eGFP fibroblasts cells stained positive for MBP on day 60 (Fig. 1h, i). Interestingly, MBP^+^/GFP^+^ cells in SOX10::eGFP fibroblasts displayed more mature branched morphology and were efficiently produced than MBP^+^/GFP^+^ cells in OLIG2::eGFP fibroblasts, this result may be due to a few SOX10^+^ cells in GFP^+^ cells of OLIG2::eGFP fibroblasts (Supplementary Fig. 4d). Taken together, these results suggest that the combination of OCT4 with small molecule cocktail (A83-01, thiazovivin, purmorphamine, VPA, and forskolin) can generate OLIG2^+^ and SOX10^+^ OPCs from human fibroblasts. Hereafter, we designate these cells as induced OPCs (OLIG2^+^ iOPCs and SOX10^+^ iOPCs, respectively) and the corresponding forskolin-containing medium as oligodendrocyte-inducing medium (OIM).

### Generation of iOPCs from human adult fibroblasts

**Fig. 2:**
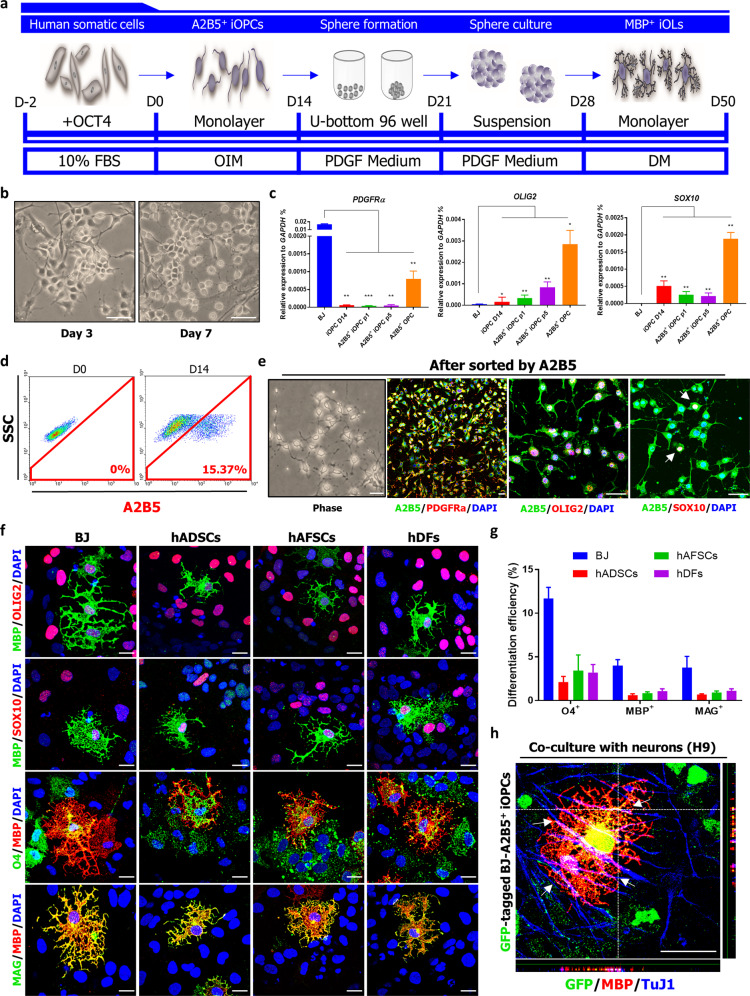
Generation of iOPCs from human somatic cells. **a** A schematic protocol for the stepwise reprogramming of human somatic cells into iOPCs and MBP^+^ Oligodendrocytes. **b** Representative images of reprogrammed BJ at day 3 and day 7 after OCT4 transduction, cultured in OIM. Scale bars, 100 µm. **c** Comparative qPCR analysis of iOPCs before and after sorting by A2B5 expression. The expression levels of the indicated genes in cells at different stages of reprogramming are shown relative to those in BJ, and the transcript levels were normalized against *GAPDH*. The data represent the mean + SD (*n* = 3). *, statistically significant difference vs. BJ cultured in OIM. *P < 0.05, **P < 0.01, ***P < 0.001 by unpaired two-tailed t-test. **d** Representative flow cytometry data for A2B5^+^ cells. The data from three independent experiments are presented as the mean values. **e** Representative fluorescence images of reprogrammed cells after A2B5 sorting. Phase-contrast images (left). The enriched expression of A2B5 was observed, and these cells coexpressed PDGFRa, OLIG2, and SOX10. The arrows denote SOX10^+^ cells. Scale bars, 100 µm. **f** Representative fluorescence images of the differentiation (3D) of A2B5^+^ iOPCs into oligodendrocytes in sphere culture. The expression of MBP with the coexpression OLIG2, SOX10, O4, and MAG was observed. Scale bars, 100 µm. **g** Quantification of O4, MBP, and MAG expression in differentiation culture on day 60. **h** GFP-tagged O4^+^ iOPCs (BJ) were cocultured with H9-hESC-derived neurons. Representative immunofluorescence images show that the MBP^+^ oligodendrocytes derived from iOPCs (GFP-tagged) can myelinate TuJ1-positive neurons in vitro. Arrows denote wrapping. Scale bars, 200 µm.

To assess whether our method could be applied to human adult fibroblasts for clinical applications, BJ were transduced with OCT4 and cultured in OIM for 2 weeks (Fig. 2a). On day 7 after transduction, OCT4-transduced BJ exhibited a bipolar morphology (Fig. 2b), on day 14, these cells expressed OLIG2 and SOX10 (Fig. 2c and Supplementary Fig. 5a) that was consistent with our observation in reporter cell lines (Fig. 1f, g). As OPCs express several cell-surface markers, including PDGFRa, NG2, and A2B5, we considered isolating OLIG2- or SOX10-positive cells from OCT4-transduced BJ using these surface markers. We observed significantly enriched A2B5^+^ populations on day 14 (Fig. 2d and Supplementary Fig. 5a) but PDGFRa (Fig. 2c) and NG2 (CSPG4) were also highly expressed in human fibroblasts, as has been observed in mouse fibroblasts^13,14^. So, we used A2B5 as the sole sorting marker and found that while most A2B5^+^ cells expressed OLIG2, SOX10 was detectable in small subsets of A2B5^+^ cells (Fig. 2e and Supplementary Table 2). These results are consistent with our reporter cell lines, in which SOX10^+^ populations were small subsets of OLIG2^+^ populations (Supplementary Fig. 4d). When cultured in a differentiation medium, the expression level of OCT4 was downregulated (Supplementary Fig. 5b) and 2.78 ± 0.53% O4^+^ cells were detectable in A2B5^+^ cells on day 40 (Supplementary Fig. 5c). MBP^+^ oligodendrocytes were also detectable on day 50 (Supplementary Fig. 5d); these were minimally branched, as known to be in immature oligodendrocytes. These results were also observed in various other human somatic cells including human adipose-derived stem cells (hADSCs), human amniotic fluid-derived stem cells (hAFSCs), and human dermal fibroblasts (hDFs) (Supplementary Fig. 5e−g). To promote maturation, we allowed A2B5^+^ cells to differentiate through sphere culture (Supplementary Fig. 6a) as performed in our previous study^25^. In comparison to 2D monolayer culture, sphere culture resulted in increased SOX10^+^ populations (Supplementary Fig. 6b) or O4^+^ (Supplementary Fig. 5c) and the resulting MBP^+^ cells exhibited a typical branched morphology with overlapping expression of OLIG2, SOX10, O4, and MAG (Fig. 2f, g). Moreover, using this approach, OLIG2^+^ iOPCs also exhibited mature MBP^+^ oligodendrocytes (Supplementary Fig. 6c), unlike in monolayer differentiation (Fig. 1h). Furthermore, these A2B5^+^ iOPCs (GFP-tagged) exhibited myelinating capacity with H9-hESC-derived TuJ1^+^ neurons (Fig. 2h and Supplementary Fig. 6d) and rat spinal cord-derived ChAT^+^ neurons^26^ (Supplementary Fig. 6e). Likewise reporter cell lines (OLIG2::eGFP and SOX10::eGFP fibroblasts) and human somatic cells, our strategy allowed for the in vitro expansion up to ten passages while retaining differentiation potential, varying with source of cell lines (Supplementary Fig. 6f−h). Hereafter, we designate A2B5^+^ and O4^+^ cells as A2B5^+^ iOPCs and O4^+^ iOPCs, respectively.

### iOPCs possess similar expression profiles as OPCs

**Fig. 3:**
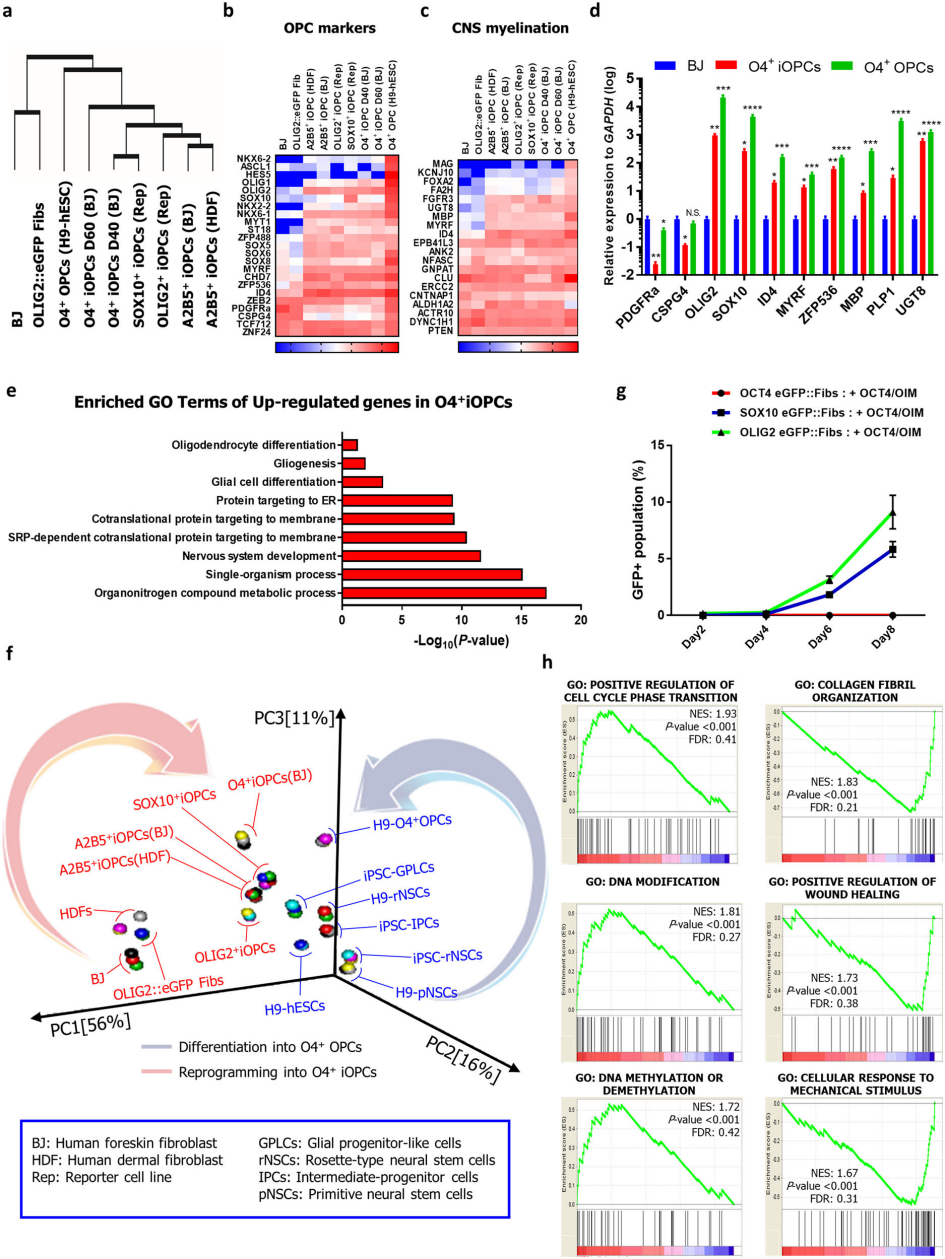
Analysis of gene expression profiles of iOPCs by RNA-seq. **a** The hierarchical clustering of 11,426 genes (FDR < 0.05) between samples from the RNA-sequencing data of A2B5^+^ iOPCs (derived from BJ and human dermal fibroblasts), OLIG2^+^ iOPCs (derived from OLIG2::eGFP fibroblasts), SOX10^+^ iOPCs (derived from SOX10::eGFP fibroblasts), O4^+^ iOPCs (derived from BJ on day 40 and 60), O4^+^ OPCs (derived from H9-hESCs on day 60), OLIG2::eGFP fibroblasts and human fibroblasts (BJ). **b**, **c** The RNA-seq profiles of gene categories are depicted as heatmaps. **d** Comparative qPCR analysis of O4^+^ iOPCs, O4^+^ OPCs, and BJ. The data represent the mean + SD (*n* = 3). *, statistically significant difference vs. BJ cultured in OIM. *P < 0.05, **P < 0.01, ***P < 0.001, ****P < 0.0001 by one-way ANOVA (N.S., not significant). **e** The enriched GO terms (biological processes) of upregulated genes in O4^+^ iOPCs relative to BJ. The *P*-value of each GO term is presented on the *x*-axis of a log_10_ scale. **f** Principal-component analysis (PCA) of the various samples mentioned in (**a**) and a previous study (Yun et al., 2019). pNSCs, primitive NSCs; rNSCs, rosette-type NSCs; IPCs, intermediate progenitor cells; GPLCs, glial restricted progenitor-like cells. **g** A quantitative representation of the flow cytometry analysis. The data from three independent experiments are presented as the mean ± SD. **h** Gene set enrichment analysis (GSEA) between a group of iOPCs and a group of fibroblasts. The group of iOPCs included A2B5^+^ iOPCs (BJ), A2B5^+^ iOPCs (HDF), OLIG2^+^ iOPCs, SOX10^+^ iOPCs, O4^+^ iOPCs (D40), and O4^+^ iOPCs (D60). The group of fibroblasts included BJ, OLIG2::eGFP fibroblasts, and HDFs.

To understand molecular changes in iOPCs, we comparatively studied the global gene expression profiles of three types of fibroblasts, six types of iOPCs, and the H9-hESC-derived O4^+^ OPCs by RNA-seq. The hierarchical clustering of a set of 11,426 genes (FDR < 0.05) revealed that several iOPCs and OPCs clustered together, separately from the two types of fibroblasts (Fig. 3a). To identify the differentially expressed genes (DEGs), we classified the genes according to previously established categories associated with oligodendrocyte development. Of note, several markers related to oligodendrocyte development and myelination, such as *OLIG2*, *SOX10*, *ID4*, *MYRF*, and *MBP*, were highly upregulated in both iOPCs and OPCs relative to fibroblasts (Fig. 3b−d). In addition, GO enrichment analysis showed that DEGs upregulated in O4^+^ iOPCs were highly enriched in processes strongly upregulated in CNS myelin (Fig. 3e)^27^. When the RNA-seq profiles of OPCs and iOPCs were compared, while iOPCs exhibited broad regional identity, OPCs prominently expressed *HOXA2*, *HOXA3*, and *HOXB4*, which represent spinal cord identity (Supplementary Fig. 7a).

To determine whether iOPCs originated from iNSCs or iPSCs rather than be directly reprogrammed since we used OCT4 as a reprogramming factor and iOPCs expressed SOX2 which is a transcription factor common to ESCs (iPSCs), NSCs, and OPCs^28^ even though our reprogramming culture conditions (OIM) were quite different from KSR-based reprogramming media used in generation of iNSCs^19,21^ and iPSCs^29^. We compared the global gene expression profiles of iOPCs to those of ESCs, NSCs, OPCs, and, glial progenitor-like cells (GPLCs) as demonstrated in our previous study^25^ by principal component analysis (PCA). Notably, we found that the progression of reprogramming from fibroblasts to iOPCs were distinct from that of differentiation (Fig. 3f) and expression profiles of iOPCs were similar to those of O4^+^ OPCs and human brain-derived oligodendrocytes^30^ (Supplementary Fig. 7b). Moreover, when NSCs and iOPCs were cultured in neuronal differentiation medium and glial differentiation medium, TuJ1^+^ neurons and glial fibrillary acidic protein (GFAP)-positive astrocytes were observed in NSC culture, but not in iOPC culture (Supplementary Fig. 7c). We also compared gene expression profiles of iOPCs with Schwann cell progenitors (SCPs) that were obtained in previous study^31^ to assess whether iOPCs have SCPs characteristics and PCA revealed that iOPCs possessed similar transcription pattern to OPCs, distinct expression profiles to SCPs (Supplementary Fig. 7d) and expression of MPZ, known as SCPs marker, was not detected in iOPC culture compared to SCP culture (Supplementary Fig. 7e). Finally, to clarify the possibility that iOPCs may gain pluripotency during the initial period of reprogramming, we analyzed the transient endogenous expression of OCT4 using the OCT4::eGFP reporter system^18^, but we did not observe any GFP^+^ cells in the OCT4-transduced cells cultured in OIM during the first week of induction (Supplementary Fig. 7f), whereas the expression of OLIG2 and SOX10 was detectable on day 6 (Fig. 3g). Accordingly, these findings suggest that although iOPCs have distinct expression profiles, they bear a high degree of similarity to OPCs and are directly reprogrammed without acquiring pluripotency or neural stemness. Furthermore, gene set enrichment analysis (GSEA) revealed a statistically significant enrichment of regulation of cell cycle-related genes, as well as of DNA modification, and methylation-related genes in six types of iOPCs compared to three types of fibroblasts (Fig. 3h). These data suggest that iOPCs induction occurred through OCT4-mediated cell division and DNA modification, prerequisite for cellular reprogramming.

### Genome-wide sequencing and analysis in generation of iOPCs

**Fig. 4:**
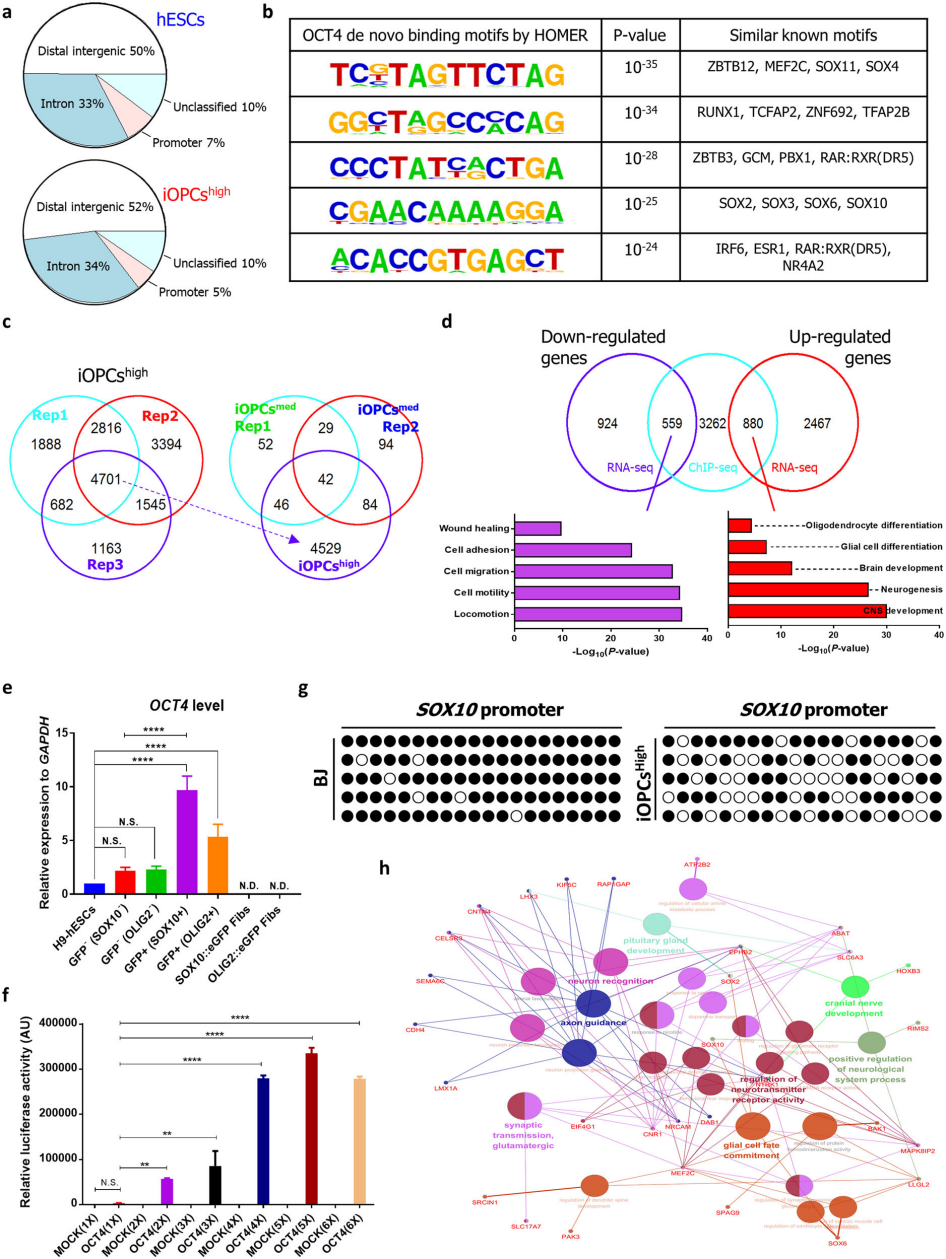
Genome-wide sequencing and analysis of OCT4 binding sites in iOPCs. **a** Annotation of OCT4 peaks as regulatory elements in H9-hESCs and A2B5^+^ iOPCs (iOPCs^high^). **b** The putative OCT-4 binding motifs that were enriched for transcription factors in A2B5^+^ iOPCs (iOPCs^high^) as determined by HOMER software. **c** A Venn diagram showing the genes containing OCT4-binding regions that overlapped among three biological replicates. **d** A Venn diagram showing the overlap of four-fold up- or downregulated genes identified by RNA-seq (right and left, respectively) in A2B5^+^ iOPCs (BJ) relative to BJ with putative OCT4 target genes (derived from ChIP-seq, middle). The *P*-values of each GO term for the overlapped genes (559 and 880) are presented on the *x*-axis of a log_10_ scale. **e** Comparative qPCR analysis of *OCT4* expression in hESCs, GFP^−^, and GFP^+^ cells. The expression is shown relative to that of H9-hESCs, and normalized to *GAPDH*. The data are represented as the mean + SD (*n* = 3). *, statistically significant difference vs. H9-hESCs. Significant differences were analyzed by one-way ANOVA. N.S., not significant; N.D., not detected. *P < 0.0001. **f** SOX10 promoter luciferase assay in fibroblasts infected with the pMXs-hOCT4 and pMXs vectors (MOCK). The level of OCT4 (1X) was comparable to that of H9-hESCs. Average values from three independent experiments are shown. *, statistically significant difference vs. OCT4 (1X). Significant differences were analyzed by one-way ANOVA. N.S., not significant. **P < 0.01, ****P < 0.0001. **g** Bisulfite sequencing analysis of the *SOX10* promoter in A2B5^+^ iOPCs (iOPCs^high^) and control BJ fibroblasts. The open circles represent unmethylated CpG sites, and the black circles represent methylated CpG sites. **h** GO term enrichment analysis of the genes associated with CNS development by ClueGO showed that genes involved in glial cell fate commitment, the regulation of neurotransmitter receptor activity, and axon guidance were significantly enriched.

OCT4, a member of the POU family, has been considered as a pioneer factor in maintaining and establishing pluripotency in ESCs and iPSCs by binding to inaccessible regions of chromatin^32^ and in the present study, DNA modification-related genes were also upregulated in OCT4-induced iOPCs (Fig. 3h). In this regard, we sought to investigate how OCT4 could induce oligodendroglial fate in fibroblasts and whether a particular level of OCT4 expression enables reprogramming using ChIP-seq in combination with RNA-seq. To analyze the genome-wide targets of OCT4 in iOPCs, we obtained DNA from H9-hESCs and fibroblasts (BJ) transduced with high levels of OCT4 (more than five-fold over an ESC level of OCT4, iOPCs^high^) or medium levels of OCT4 (comparable to an ESC level of OCT4, iOPCs^med^) subjected to ChIP with anti-OCT4 antibody (at day 14 after OCT4 transduction, sorted by A2B5 expression) with different batches of the OCT4 virus. Our analysis of ChIP-seq data identified that the majority of OCT4-binding regions correlated with distal regulatory elements both hESCs and iOPCs^high^ (Fig. 4a), consistent with the previous report^32^. The OCT4-binding sites in hESCs were highly enriched for known OCT4-binding motifs, including CTCF, OCT4, and CTF (pluripotent-related genes), while those in iOPCs^high^ were not (Supplementary Fig. 7g). Interestingly, the putative de novo OCT4-binding motifs in iOPCs^high^ were enriched in DNA elements for transcription factors, including the SOX family (Fig. 4b). We selected 4701 genes correlated with OCT4-binding sites (FDR < 10^−5^) in iOPCs^high^ (Fig. 4c). Among the 4701 genes, 880 genes were upregulated and 559 genes were downregulated in iOPCs^high^ compared to fibroblasts; GO analysis showed that the upregulated genes were involved in the development of CNS, while the downregulated genes were involved in wound healing, which is representative of the genes expressed in fibroblasts (Fig. 4d). The upregulated genes included *ID4*, *NKX6.1*, *OLIG2*, *PTPRZ1*, *SOX2*, and *SOX11*, which are key factors in oligodendrocyte development (Supplementary Fig. 7h). In contrast, medium levels of OCT4 exhibit low binding affinity for that of such genes in iOPCs^med^ (Fig. 4c). In addition, we found that the endogenous expression of OLIG2 and SOX10 was consistent with high levels of OCT4 (Fig. 4e). Further analysis revealed that *SOX10* promoter activity was highly correlated with the dose of OCT4 (Fig. 4f) and CpG sites of the *SOX10* promoter were demethylated in iOPCs^high^ as opposed to fibroblasts (Fig. 4g)^33^.

Taken together, these data suggest that high levels of OCT4 may reconstruct a new functional gene regulatory network involved in CNS development and induce cell fate conversion by modulating these genes (Fig. 4h).

### Therapeutic efficacy of iOPCs in MOG-induced EAE

**Fig. 5:**
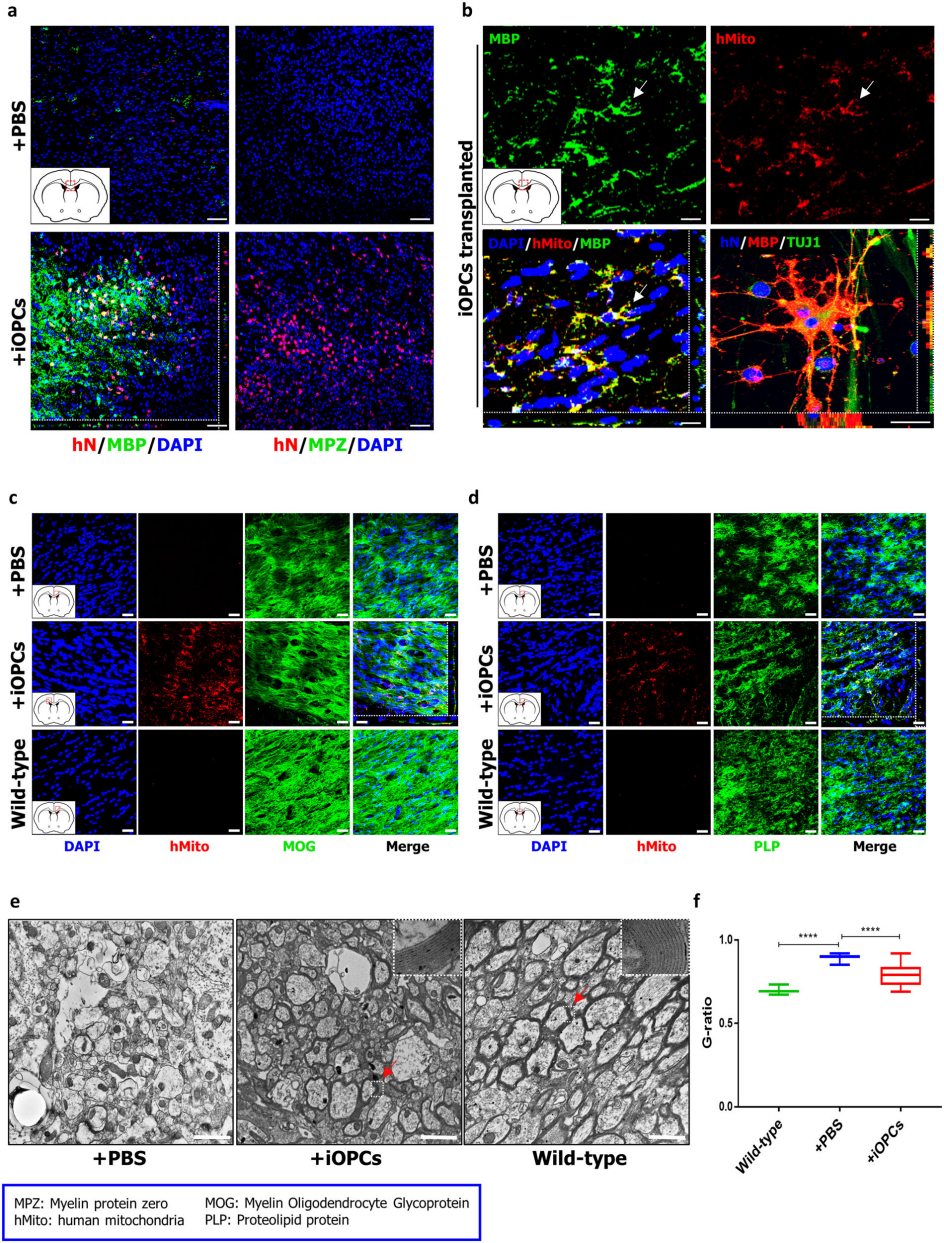
Analysis of remyelination upon transplantation of iOPCs in shiverer mice. **a** Shiverer mice were implanted in the corpus callosum with O4^+^ iOPCs or treated with PBS. The transplantation of iOPCs into the corpus callosum resulted in their differentiation into MBP-positive oligodendrocytes. Transplanted iOPCs were identified using a human-specific nuclear antibody. Scale bars, 100 µm. Higher-magnification fluorescence images of MBP- (**b**), MOG- (**c**), and PLP- (**d**) positive engrafted cells. Engrafted iOPCs were identified using a human-specific anti-mitochondrial antibody. Scale bars, 20 µm. **e** Representative electron microscope images of PBS-treated brains of shiverer mice, iOPC- engrafted brains of shiverer mice, and untreated brains of wild-type mice. While the engrafted corpus callosum in iOPC-transplanted and wild-type brains show compact myelin with major dense lines, the PBS-treated brains contained thin myelin sheaths. The samples of the corpus callosum were obtained 14 weeks after engraftment. The arrows denote the original position of magnified picture. Scale bars, 2 µm. **f** The comparison of the g-ratio in the corpus callosum among the PBS-treated brains of shiverer mice, iOPC- engrafted brains of shiverer mice, and untreated brains of wild-type mice. The graph represents the g-ratio obtained from axons with a diameter >1 µm and was calculated from three mice per group (*n* = 100). *, statistically significant difference vs. PBS-treated group. Significant differences were analyzed by one-way ANOVA (iOPCs, ****P < 0.0001; Wild-type, ****P < 0.0001).

Next, we examined whether iOPCs could myelinate axons in congenitally hypomyelinated shiverer mice. Four-week-old homozygous shiverer (shi/shi) mice were implanted in the corpus callosum with 500,000 O4^+^ iOPCs or treated with PBS as previously described^25^. Both groups of mice received cyclosporine-A daily starting the day before transplantation. Following 14 weeks of engraftment, the brains of sacrificed mice were analyzed to examine the competence for myelination by the engrafted iOPCs. Notably, we observed MBP^+^ cells in the corpus callosum of the iOPC-engrafted brains that were positive for markers of both human nuclei and human mitochondria, while the control PBS-treated brains did not exhibit any expression of MBP (Fig. 5a, b). Moreover, the cells positive for markers of human mitochondria also coexpressed MOG and PLP1 (Fig. 5c, d). Furthermore, transmission electron microscopy (TEM) of the iOPC-engrafted brains revealed compact myelin with major dense lines, comparable to that of non-diseased mice, whereas the brains of untransplanted shiverer mice did not exhibit such lines (Fig. 5e). The g-ratios also suggested extensive remyelination in the engrafted brains (Fig. 5f). These results demonstrate that iOPCs can differentiate into myelinating oligodendrocytes in vivo.

**Fig. 6:**
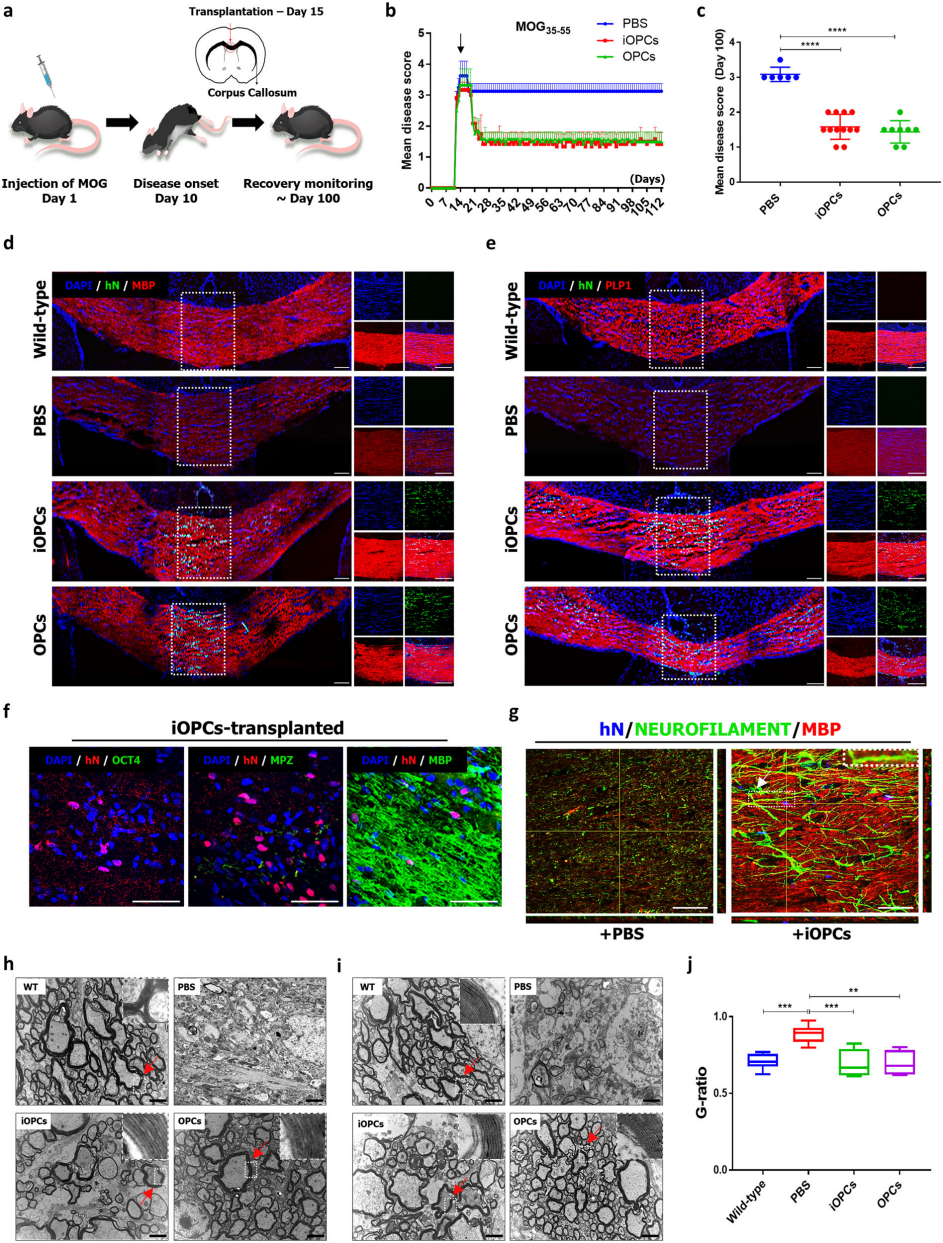
iOPCs alleviate disease severity in an MOG-induced EAE model. **a** A schematic overview of iOPC transplantation in an MOG-induced EAE mouse model. **b**, **c** The disease severity scores of PBS-treated, iOPCs- engrafted, and OPCs- engrafted MOG-induced EAE mice over 100 days. OPCs and iOPCs were engrafted on day 15 after MOG-induced disease induction (black arrow). The values represent the mean ± s.e.m.; *n* = 6–12 mice per group. Significant differences were analyzed by one-way ANOVA. ****P < 0.0001. **d**, **e** Representative fluorescence images on day 100 post-disease induction. The right panels represent multichannel immunofluorescence images of the boxed areas in the corresponding left panels. Scale bars, 100 µm. **f** Representative fluorescence images 100 days after transplantation. Scale bars, 100 µm. **g** Representative fluorescence images of in vivo myelination. The boxed area in the image on the right represents a magnified (3×) picture of neurons myelinated by iOPC-derived oligodendrocytes (white arrow). Scale bars, 30 µm. Representative electron microscope images of PBS-treated brains of EAE mice, iOPCs- engrafted brains of EAE mice, OPCs- engrafted brains (**h**) and spinal cord (**i**) of EAE mice, and untreated brains of wild-type mice. The samples were obtained 14 weeks after engraftment. The arrows denote the original position of magnified picture. Scale bars, 2 µm. **j** The comparison of the g-ratios among the PBS-treated brains of shiverer mice, iOPC- engrafted brains of shiverer mice, OPC- engrafted brains of shiverer mice, and untreated brains of wild-type mice. The graph represents the g-ratio obtained from axons with a diameter >1 µm. *, statistically significant difference vs. PBS-treated group. Significant differences were analyzed by one-way ANOVA. **P < 0.01, ***P < 0.001.

Given a beneficial potential of iOPCs as a remyelinating therapeutic agent, we next determined whether iOPCs could effectively promote remyelination in a demyelinating disease model. We used a mouse model of MOG_35–55_-induced EAE, which recapitulates chronic progressive demyelination^34^ (Fig. 6a). After 15 days of immunization with MOG_35–55_ (at the peak of disease score), EAE mice were randomly divided into three groups that received PBS or were engrafted with O4^+^ iOPCs or hESC-derived O4^+^ OPCs (*n* = 14 mice per group) in the corpus callosum (Supplementary Fig. 8a). Monitoring and scoring of the engrafted mice for over 100 days (Supplementary Table 3) revealed that both groups of mice that received iOPCs and those that received OPCs exhibited similar alleviation of disease symptoms and considerable improvement in disease score within 10 days, whereas the PBS-treated mice exhibited only partial recovery with persisting functional deficits (Fig. 6b). Moreover, we observed a significant remyelination in the corpus callosum and spinal cord of mice transplanted with OPCs or iOPCs, whereas PBS-treated mice exhibited a poor myelination (Supplementary Fig. 8b−d). The statistical analysis of disease scores on day 100 after engraftment showed that the transplantation of OPCs or iOPCs resulted in extensive functional recovery to levels comparable to normal mice (Fig. 6c and Supplementary Movie 1). There were no aberrant signs such as tumor formation in the brains and spinal cord engrafted with either iOPCs or OPCs (Supplementary Fig. 8e). Indeed, we observed that the engrafted OPCs and iOPCs survived beyond 100 days after transplantation, integrated into the host nervous system, and differentiated into fully mature MBP^+^ (Fig. 6d and Supplementary Fig. 8f), PLP1^+^ (Fig. 6e and Supplementary Fig. 8f), and MOG^+^ oligodendrocytes (Supplementary Fig. 8g, h). In addition, survived cells migrated toward lesions in lumbar spinal cord^35^ and there were no MPZ^+^ or OCT4^+^ cells among the survived cells suggesting engrafted iOPCs solely differentiated into oligodendrocytes (Fig. 6f). Furthermore, the engrafted iOPCs were capable of remyelinating the axons of host neurons (Fig. 6g), strongly supporting the significant reduction in clinical severity upon remyelination; these cells were widely distributed along the white matter tracts^36^, demonstrating the efficient migration and colonization of iOPCs that is crucial to therapy (Supplementary Fig. 9a−d). Given that OPCs can differentiate into astrocytes in vivo, we also assessed GFAP expression and found few GFAP^+^ astrocytic cells (Supplementary Fig. 9a) as previously observed^9,15^. Next, we used TEM to validate that the engrafted iOPCs could regenerate myelin sheaths. TEM of iOPC- and OPC-transplanted mouse brains and spinal cords showed abundant compact myelin sheath with major dense lines, whereas PBS-treated brains showed poor remyelination (Fig. 6h, i). The g-ratios of the myelin produced by iOPCs and OPCs were indistinguishable from that in wild-type mice but were significantly lower than that in PBS-treated mice (Fig. 6j). Collectively, these data strongly suggest that iOPCs have therapeutic efficacy in demyelinating diseases by integrating into the host nervous system.

## Discussion

**Fig. 7:**
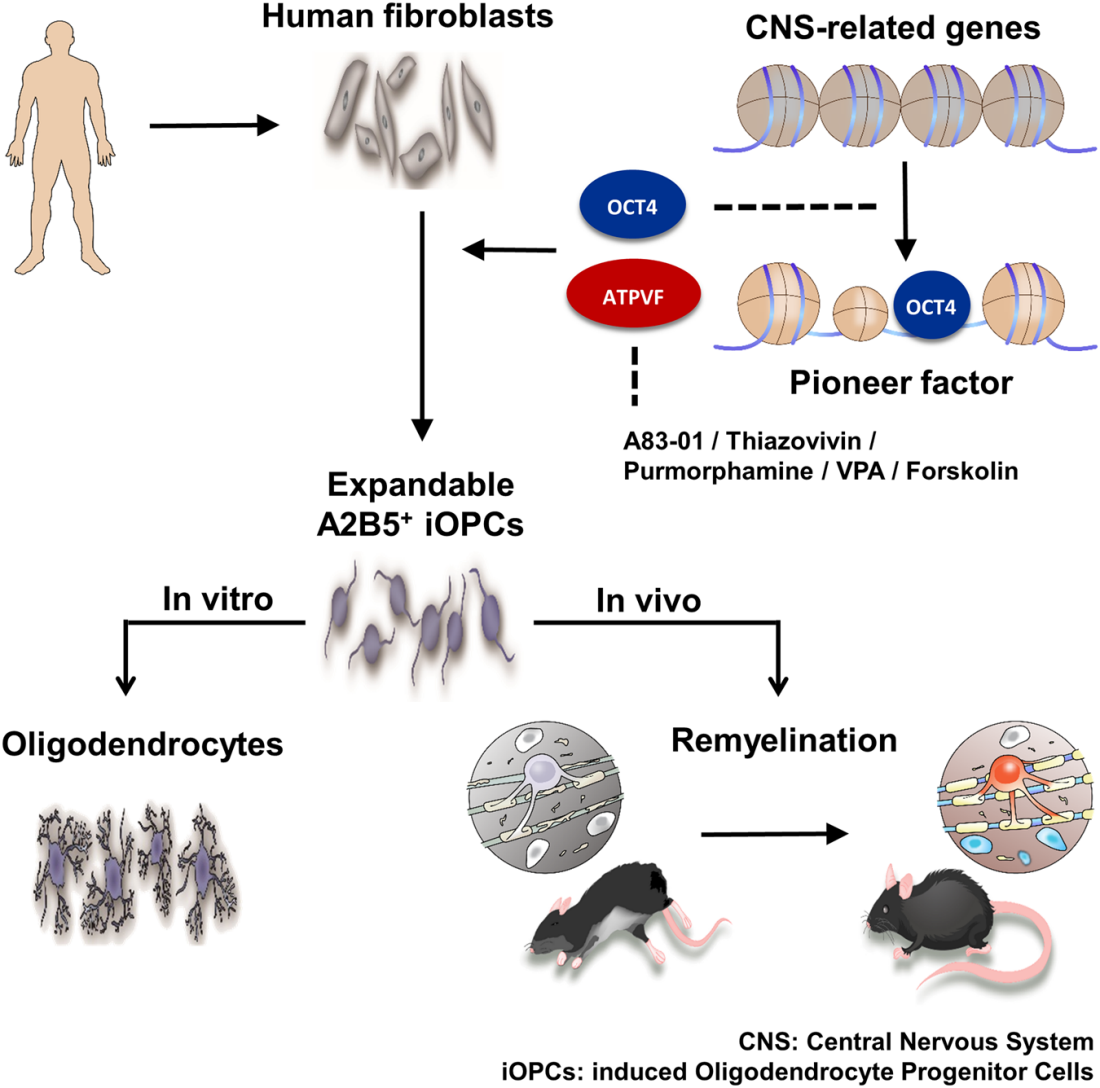
Schematic summary of the present study. This study describes the role of OCT4 in combination with defined small molecules for direct reprogramming of human somatic cells into oligodendrocyte progenitor cells (OPCs). The expandable and transplantable induced OPCs (iOPCs) promoted remyelination in shiverer mice with hypomyelination and rescued the disease phenotype in a mouse model of multiple sclerosis.

OCT4, a master regulator of pluripotency, has been proposed as a lineage specifier that can be regulated by altering experimental conditions^37,38^. Similarly, recent studies have reported the transcription factor-driven conversion of mouse somatic cells into iOPCs in vitro^39,40^ and OPCs like cells in vivo^41,42^, suggesting that OCT4 is sufficient to induce oligodendroglial lineage in human somatic cells and that lineage decisions may rely on the expression level of OCT4 with cell-extrinsic cues^38,43^. In the present study, our findings clearly indicate that the ectopic expression of OCT4 in combination with small molecules, governing cell fate options and oligodendrocyte development such as A83–01 (inhibitor of transforming growth factor β kinase type 1 receptor)^44^, thizovivin (rho kinase [ROCK] inhibitor)^45^, VPA (histone deacetylase inhibitor)^41^. purmorphamine (sonic hedgehog [SHH] agonist)^6^, and forskolin (cyclic adenosine monophosphate [cAMP] activator)^24^, allows the completion of the direct reprogramming of human fibroblasts into A2B5^+^ iOPCs expressing OLIG2 and SOX10 within two weeks (Fig. 7). The upregulation of SOX10 is known as an indicator of the successful reprogramming into cells of oligodendroglial lineage^16,46,47^. However, there are less SOX10^+^ populations in our A2B5^+^ iOPCs since A2B5 comprised a small proportion of OPCs in human brains unlike rodent brains^48^, which resulted in less pure populations and low differentiation efficiency. Nonetheless, isolation of iOPCs in OCT4-transduced human adult fibroblasts is currently reliant on the expression of A2B5 (usable surface marker not shared with fibroblasts). Moreover, it is becoming evident that OPCs are functionally heterogeneous depending on brain regions, ages, and pathology^49–51^. Alternative surface markers or clinically applicable strategies for identifying pure OPCs would allow us to better understand cell fate conversion and obtain more pure populations exhibiting OPC characteristics in iOPCs-culture.

O4^+^ iOPCs exhibited similar molecular features as OPCs in terms of the transcriptome and differentiation potential but there are some genes expressed unique to iOPCs, even though the global gene expression profiles of iOPCs seem to be more similar to those of OPCs than to those of fibroblasts. One possible explanation is the broad targeting ability of OCT4 since this gene is not expressed in bona fide OPCs. Another possible explanation is degree of reprogramming depending on levels of OCT4 since, in iOPCs^high^, numerous OCT4 binding sites occupied regulatory regions of up-regulated DEGs (CNS development-related genes) and down-regulated DEGs (fibroblast-specific transcriptional network) but not in iOPCs^med^. Our current protocol is largely based on overexpression (more than five-fold over an ESC level of OCT4) and whether OCT4 directly regulates these DEGs or plays a central role in chromatin accessibility as the pioneer factor^32^ remains uncertain but these results suggest that the proper expression level of OCT4 is required for successful reprogramming. Alternatively, the introduction of lineage-specific or relevant transcription factors, a corresponding to downstream targets of OCT4, may lead to more refined reprogramming, comparable to that of bona fide OPCs.

The limited natural remyelination mediated by resident oligodendroglial progenitors has led to efforts in understanding demyelination and the regenerative process of remyelination. Although these efforts have paved the way for a broad range of options to treat CNS myelin disorders, including MS^52^, none of the currently available treatments directly contribute to controlling the progression and repair of myelin disorders, either acquired or inherited^53^. In the brains of EAE mice as a representative model for MS, we demonstrated that the generated iOPCs could integrate into the host nervous system, differentiate into mature oligodendrocytes, ameliorate disease symptoms to levels comparable to wild-type mice, and exhibit no tumorigenic effect; but there are still potential risks for tumorigenesis by the usage of OCT4. Alternatively, lineage transcription factor-driven reprogramming^15^ would be more safe when it governs successful iOPCs-generation in human fibroblasts bypassing the pluripotent iPSC stage. In addition, small molecules-based reprogramming still remains challenging for future clinical application. Combinatorial small molecules have been used to enhance reprogramming efficiency or replace some of the transcription factors. Current strategies require activation of chromatin and modulation of the specific signaling pathway for the cell fate conversion but the combination of small-molecules affect a wide range of gene expression, which might lead to incomplete reprogramming or reduce the availability in different environment such as clinical laboratories. More efforts such as minimizing multiple usage of small molecules by identifying novel compounds are required to establish the refined reprogramming and more widely applicable methods.

For clinical applications, further studies on improvement of iOPCs identity, the deeper understanding role of OCT4, expansion of CNS myelination, long-term functional benefits to transplant recipients, and nonintegrating genetic delivery to avoid potential issues associated with viral integration are required. Nevertheless, this study proposes a novel method for generating expandable human iOPCs, which may facilitate their broad applicability in a rapid, efficient, and patient-specific manner. In addition, these cells may prove useful for pharmacological screenings and as an in vitro platform for studying human myelin diseases of the brain and spinal cord at the molecular and cellular levels.

## Methods

### Cells

H9-hESCs (WA09), provided by WiCell Research Institute (Madison, WI, USA); SOX10::eGFP reporter hESCs, provided by Dr. Gabsang Lee (Johns Hopkins University, Baltimore, USA); OLIG2::eGFP reporter hESCs (R‐Olig2), provided by Dr. Ying Liu (University of Texas, Austin, USA). BJ (human foreskin fibroblasts) and HDFs (human dermal fibroblasts), obtained from ATCC (Manassas, VA, USA); hADSCs (human adipose-derived stem cells), obtained from Biostar Stemcell Research Institute (Seoul, South Korea), hAFSCs (human amniotic fluid-derived stem cells) obtained from our previous study^54^. Differentiation of NSCs^25^, O4^+^ OPCs^9^, and CD49d^+^ SCPs^31^ in H9-hESCs were performed as previously described.

### Cell culture

SOX10::eGFP and OLIG2::eGFP reporter hESCs were maintained in E8 medium on dishes coated with Matrigel (BD Biosciences Clontech, Palo Alto, CA, USA) and differentiated into fibroblasts using Dulbecco’s Modified Eagle’s Medium (DMEM, HyClone/Thermo Fisher Scientific, Waltham, MA, USA) supplemented with 10% fetal bovine serum (FBS, HyClone) and 1% penicillin-streptomycin (Thermo Fisher Scientific) as previously described^18^. All human fibroblast lines, including BJ, SOX10::eGFP fibroblasts, OLIG2::eGFP fibroblasts, ADSCs, AFSCs, and DFs, were cultured in the above-mentioned medium before transduction.

### Generation of high-titer retrovirus

The coding sequences (CDS) of OLIG2, SOX10, NKX6.2, NKX2.2, ID2, and ID4-derived from total human spinal cord cDNA or hESC-derived pre-OPCs (generated by previous study, Yun et al., 2019) were cloned to pMXs-vectors. pMXs retroviral vectors (including OCT4, SOX2, BMI1, and GFP) were transfected into the Platinum-GP cell line (Cell Biolabs, Inc., San Diego, CA, USA) using Lipofectamine 2000 (Invitrogen, Waltham, MA, USA) according to the manufacturer’s protocol. The viruses were collected at 72, 96, 120, 144, and 168 h using DMEM supplemented with 10% FBS and filtered through a 0.45-μm filter. Forty milliliters of the harvested virus was then concentrated by ultracentrifugation (Beckman Abanti J-E, Beckman Instruments, Palo Alto, CA, USA) at 20,000 × *g* for 2 h. After centrifugation, the supernatant was carefully decanted, and the obtained virus particle pellet was resuspended in 2 ml of cooled DMEM and stored at 4 °C overnight. The obtained viruses were used each day without being stored further.

### Induction of oligodendrocyte progenitor cells

Human fibroblasts were seeded at 2 × 10^5^ cells per well in a 6-well plate and transduced the next day with 1 ml of concentrated retrovirus (containing more than 15 ml of virus soup) and 4 μg/ml polybrene (Sigma-Aldrich, St. Louis, MO, USA). To increase the transduction efficiency, spin-infection was carried out by centrifugation at 3000 × *g* for 30 min at room temperature during the initial time of infection. Following transduction for 6 h, the medium was replaced with fresh growth medium.

Two days after transduction, the cells were detached using Accutase (EMD Millipore, Burlington, MA, USA) and seeded at 1 × 10^5^ cells/well in poly-l-ornithine/laminin-coated six-well plates and cultured in oligodendrocyte inducing media (OIM) consisting of DMEM supplemented with 1 × B-27 supplement without vitamin A (Thermo Fisher Scientific), 1× N-2 Supplement (Thermo Fisher Scientific), 1% penicillin–streptomycin, 1% L-glutamine (Thermo Fisher Scientific), 1% nonessential amino acids (Thermo Fisher Scientific), 20 ng/ml FGF2 (Peprotech, Rocky Hill, NJ, USA), 20 ng/ml PDGF-AA (Peprotech), 50 μg/ml ascorbic acid (Sigma Aldrich), 0.5 μM A-83-01 (Tocris, Bristol, UK), 0.5 μM thiazovivin (Tocris), 250 μM valproic acid (VPA, Sigma Aldrich), 0.5 μM purmorphamine (Tocris), and 10 μM forskolin (Millipore) for two weeks.

### In vitro differentiation into oligodendrocytes

For in vitro differentiation, two weeks after transduction, A2B5^+^ iOPCs were purified by MACS (magnetic-activated cell sorting, Miltenyi Biotec) according to the manufacturer’s instructions; 5,000 cells were plated in each well of an ultra-low binding 96-well plate, suspended in PDGF medium consisting of DMEM/F12 (3:1 mixture) supplemented with 1 × B-27 Supplement without vitamin A, 1 × N-2 Supplement, 1% penicillin–streptomycin, 1% L-glutamine, 1% nonessential amino acids, 20 ng/ml PDGF-AA, 10 ng/ml IGF-1 (Peprotech), 10 ng/ml HGF (Peprotech), 10 ng/ml NT-3 (Peprotech), 10 μM forskolin, 60 ng/ml 3,3′,5-triiodo-L-thyronine (T3; Sigma-Aldrich), and 10 μg/ml insulin (Sigma-Aldrich) for 2 weeks; seeded in poly-l-ornithine/laminin-coated plates and cultured in differentiation medium (DM) consisting of DMEM/F12 (3:1 mixture) supplemented with 1 × B-27 Supplement without vitamin A, 1 × N-2 supplement, 1% penicillin–streptomycin, 1% L-glutamine, 1% nonessential amino acids, 60 ng/ml T3, 10 μM forskolin, and 50 μg/ml ascorbic acid for 1 month. O4^+^ iOPCs were also purified by MACS on day 40 and 60 after OCT4 transduction.

### Chemical screening

OCT4-transduced SOX10::eGFP and OLIG2::eGFP fibroblasts were seeded at 4 × 10^4^ cells/well in poly-l-ornithine/laminin-coated 12-well plates and were incubated with ATPV-based medium containing each of the small molecule candidates for one week; expression of GFP was analyzed by flow cytometry. To determine the additional effect of each of small molecules, 5 μM EX-527 (Tocris), 2 μM (±)-BayK 8644 (Sigma-Aldrich), 0.5 μM RG108 (Tocris), 1 μM DMH1 (Tocris), 0.1 μM Retinoic acid (Sigma-Aldrich), 10 μM forskolin, 2 μM lysophosphatidic acid (LPA, Tocris), 2 μM Parnate (Tocris), 1 μM Dexamethasone (Sigma-Aldrich), and 3 μM CHIR99021 (Tocris) were added in ATPV-based medium, consisting of OIM devoid of forskolin.

### RT-PCR and qRT-PCR analyses

For RT-PCR and qPCR, RNA was prepared from samples using TRIzol (Thermo Fisher Scientific) and cDNA was generated using Reverse Transcriptase II (Invitrogen). qPCR was conducted using the iCycler iQ (Bio-Rad) and reactions were performed using SYBR Green PCR Master Mix (Bio-Rad). Negative controls included a reverse transcription-negative blank of each sample and a no-template blank. The gene expression levels were normalized to the corresponding level of *GAPDH*, which was used as an internal control. The primers used for RT-PCR and qPCR are listed in Supplementary Table 4.

### Genomic DNA methylation analysis

Genomic DNA derived from BJ and A2B5^+^ iOPCs was isolated using the Genomic DNA Purification Kit (Promega, USA), and the bisulfite conversion of genomic DNA was performed by using the EpiTect Bisulfite Kit (Qiagen, Hilden, Germany). The resulting fragments were cloned using the pGEM-T Easy Vector (Promega) for sequencing and sequenced with T7 forward and SP6 reverse primers. The primers used for promoter fragment PCR amplification are listed in Supplementary Table 4.

### Immunostaining

Cells were fixed for 10 min at room temperature in 4% paraformaldehyde, rinsed three times with phosphate-buffered saline (PBS), and then permeabilized for 15 min at room temperature in PBS containing 0.2% Triton X-100. The fixed, permeabilized cells were blocked with 5% normal donkey serum and 0.01% Triton X-100 for 1 h and then incubated with the appropriate primary antibodies overnight at 4 °C. To label A2B5 and O4, nonpermeabilized cells were incubated with the appropriate antibody. Following primary antibody exposure, the cells were washed three times with PBS and incubated with the appropriate fluorescence-conjugated secondary antibody at a dilution of 1:500 for 1 h at room temperature. The nuclei were counterstained with 1 μg/ml DAPI (Sigma-Aldrich) for 5 min, and the samples were rinsed three times with PBS. Images were obtained with an Olympus 1×81 inverted fluorescence microscope.

For the immunostaining of the brains, the samples were embedded in O.C.T. compound (Tissue Tek, Sakura Finetek U.S.A., Inc., Torrance, CA, USA) and cryosectioned at a thickness of 10 µm. The sections were blocked with 2% normal donkey serum and 0.2% Triton X-100 in PBS for 1 h, incubated overnight at 4 °C with the indicated primary antibodies, and then incubated at room temperature for 1 h with the appropriate Alexa Fluor 488- or 594-conjugated secondary antibodies (Thermo Fisher Scientific). The nuclei were counterstained with 1 μg/ml DAPI (Sigma-Aldrich) for 5 min. The samples were rinsed three times with PBS and observed under an Olympus confocal laser scanning microscope. The antibodies used for immunostaining are listed in Supplementary Tables 5 and 6.

### Flow cytometry

To analyze the expression of GFP, A2B5, and O4, cells were harvested with Accutase, rinsed three times with cold PBS, blocked with 5% normal donkey serum for 15 min, stained with the appropriate primary antibodies for 30 min, incubated with fluorescence-conjugated secondary antibodies for 30 min, and then fixed with 0.5% PFA. All flow cytometry analyses were performed on a FACS Verse flow cytometer (BD Biosciences, San Jose, CA, USA). To isolate GFP^+^ cells from SOX10::eGFP fibroblasts and OLIG2::eGFP fibroblasts, the induced cells were digested with Accutase and washed with FACS buffer, and the cell suspensions were filtered through a 45 μm strainer. The GFP^+^ cells were sorted using a BD FACSAriaII system (BD Biosciences).

### RNA-seq data analysis

Total RNA extraction from OLIG2::eGFP fibroblasts, human dermal fibroblasts, A2B5^+^ iOPCs (BJ), A2B5^+^ iOPCs (HDFs), O4^+^ iOPCs (BJ, 2D), OLIG2^+^ iOPCs, and SOX10^+^ iOPCs was performed using TRIzol (Thermo Fisher Scientific), and genomic DNA was removed by DNase I. RNA quality was checked with an Agilent 2100 bioanalyzer (RNA Integrity Number [RIN] > 8). cDNA libraries were constructed using the TruSeq RNA Library Prep Kit (Illumina, San Diego, CA, USA) by Teragen Etex Bio Institute (Seoul, South Korea), and the libraries were sequenced on an Illumina HiSeq 2500 system. The gene expression levels were measured with Cufflinks v2.1.1 using the latest release of the Ensembl gene annotation database. To improve the accuracy of the measurements, the multiread correction and fragbias-correct options were applied; all other options were set to the default values. Transcriptome datasets used in the PCA were obtained from GSE117664 (Yun et al., 2019, PSCs-derived differentiated cells from hESCs and hiPSCs) and GSE73721 (Zhang Y et al., 2016, human brain-derived cells). Since hierarchical clustering exhibited clustered in accordance with study or platform, bias-corrected expression was carried out for data comparison. The DEGs were identified using Cuffdiff with a false discovery rate (FDR) < 0.05 and further screened by 2- or 4-fold changes of fragments per kilobase of transcript per million mapped reads (FPKM) in at least one sample of the group. The RNA-seq data are available from the NCBI GEO database (accession number GSE130063).

### ChIP-seq data analysis

To determine the dose-dependent effect of OCT4, two types of viral concentration (high and medium were employed into fibroblasts. H9-hESCs and fibroblasts (BJ) transduced with high levels of OCT4 (more than 5-fold over an ESC level of OCT4, iOPCs^high^) or medium levels of OCT4 (comparable to an ESC level of OCT4, iOPCs^med^) were cross-linked with 1% (wt/vol) formaldehyde fixation buffer (at day 14 after OCT4 transduction, sorted by A2B5 expression), resuspended and lysed using an EZ-ChIP kit (Millipore). To shear the cross-linked DNA, the lysates were subjected to 30 cycles of 30 s of sonication followed by 30 s of rest on ice with a Bioruptor sonicator (Diagenode, UCD-200) and incubated with protein G agarose beads and 3 μg of an anti-OCT4 antibody overnight at 4 °C. After chromatin immunoprecipitation, the rest of the experiments were performed according to the manufacturer’s protocols. The library was constructed using the NEBNext UltraTM DNA Library Prep Kit for Illumina (New England Biolabs, UK) according to the manufacturer’s instructions. Briefly, the ChIPped DNA was ligated with adaptors. After purification, PCR was performed with adaptor-ligated DNA and an index primer for multiplexing sequencing. The library was purified using magnetic beads to remove all of the reaction components. The size of the library was assessed by an Agilent 2100 bioanalyzer (Agilent Technologies, Amstelveen, Netherlands). High-throughput sequencing was performed as single-end 75 sequencing using NextSeq 500 (Illumina, Inc., USA). The reads were trimmed and aligned using Bowtie2^55^. Bowtie2 indices were either generated from the genome assembly sequence or the representative transcript sequences for aligning to the genome or the transcriptome, respectively. We used MACS (model-based analysis of ChIP-seq) to identify the peaks from the alignment file and referred to published ChIP-Seq datasets GSE69646 of OCT4 in hESCs. Gene classification was based on searches performed by DAVID (http://david.abcc.ncifcrf.gov/) and MEDLINE databases (http://www.ncbi.nlm.nih.gov/). The ChIP-seq data are available from the NCBI GEO database (accession number GSE130565).

### Functional gene analysis using Cytoscape

The protein interaction network was presented using Cytoscape (3.7.1) software. The differentially up-regulated genes correlated with OCT4-binding sites in iOPCs were selected to visualize the protein interaction network. To identify the molecular mechanisms of each cluster, we utilized ClueGO (2.5.5) software in the Cytoscape, which delivers a fundamentally organized protein network. GO terms (DAVID Tool) were represented as nodes and significantly (*p*-values < 0.05) enriched pathway was presented among each related genes in nervous system and glial development.

### Luciferase promoter-reporter assay

The promoter-reporter assay was performed using the Pierce Gaussia Luciferase Flash Assay Kit (Thermo Fisher Scientific). The lentiviruses containing the promoter-reporter clone of human *SOX10* promoter (GeneCopoeia; cat.HPRM47009-LvPG02) were generated from 293FT cells using lipofectamine 2000. BJ (human foreskin fibroblasts) were transduced with the lentiviruses, selected using 2 µg/ml puromycin for three days, and then transduced the next day with retroviruses containing OCT4 (pMXs-hOCT4) or empty vector (pMXs vector, Mock). Luciferase activities were measured on a Multi-Detection Microplate Reader (HIDEX) on day 7 after transduction, relative to cells transduced with control empty vector (Mock). To determine the dose-dependent effect of OCT4, a wide range of viral concentration was employed.

### Mice

A total of 50 female mice (Eight-week-old C57BL/6) were purchased from OrientBio (Seoul, South Korea) and acclimated for 11 weeks. The randomly selected 42 female mice were immunized subcutaneously with an emulsion of MOG_35–55_ in complete Freund’s adjuvant (CFA) and were then administered pertussis toxin (PTX) (Hooke Kit TM MOG_35–55_/CFA Emulsion PTX) on the day of immunization and then again the following day, as described by the manufacturer. EAE onset was monitored daily and scored on a scale of 1–5 by the following established standard criteria: score 0, no observable disease; score 0.5, limpness of the tip of the tail; score 1, tail limpness; score 1.5, tail limpness and hind leg inhibition; score 2, limpness of the tail and partial limb weakness; score 2.5, limpness of the tail and dragging of the hind legs; score 3, paralysis of one hind limb; score 3.5, paralysis of both hind limbs; score 4, paralysis of both hind limbs and partial paralysis of the front legs; score 4.5, paralysis of both hind limbs and partial paralysis of the front legs with no movement around the cage; score 5, moribundity/death. Once the mice reached the peak of disease (~day 14; clinical score = 3.5), they were randomized into three groups (PBS-treated, iOPCs-engrafted, and OPCs-engrafted mice, *n* = 14 mice per group). The experimenters who assessed and scored the mice daily were blinded to the identity of the animals. These mice were sacrificed 14 weeks after transplantation and the brains and spinal cords were analyzed to examine the competence for myelination by the engrafted cells.

All animal experiments including animal care and safety were performed with strict adherence to the guidelines of the Institutional Animal Care and Use Committee of Korea University (Seoul, South Korea, approval no. KUIACUC-2015-168). All animal surgery was performed under deep anesthesia using a combination of ketamine (100 mg/kg) and xylazine (10 mg/kg).

### Transplantation of MOG_35–55_-induced EAE mice and shiverer mice

All animals, including the PBS-treated group, received 5 mg/ml cyclosporine daily starting the day before transplantation. O4^+^ OPC and iOPC suspensions were prepared at a concentration of approximately 200,000 cells/µl. The cell suspensions (5 µl) were transplanted over a period of 5 min bilaterally to the cerebral ventricles (Coordinates: AP = +0.9 mm, ML = 0.3 mm, DV = −2.8 mm) of the anesthetized animals 15 days after EAE onset. The cells were delivered by a Hamilton syringe (Hamilton, Reno, NV, USA) attached to a stereotaxic frame (David Kopf Instruments, Tujunga, CA, USA). Animals that received the same surgery with an injection of 5 µl of PBS served as controls. To validate their myelination capacity, 500,000 O4^+^ iOPCs (*n* = 6) or OPCs (*n* = 6) were transplanted into the corpus callosum (coordinates: AP = +0.9 mm, ML = 0 mm, DV = −2.8 mm) of 4-week-old homozygous shiverer mice (The Jackson Laboratory, Bar Harbor, ME, USA) using the same procedure. Before transplantation, the mice were randomized into three groups (PBS-treated, iOPCs-engrafted, and OPCs-engrafted mice, *n* = 6 mice per group)

### Luxol fast blue staining

Cryosectioned brains were defatted in 1:1 alcohol/chloroform (Carlo Erba Reagents Srl, Via Merendi, Italy) overnight and were then incubated in Luxol Fast Blue solution (GeneCopoeia, Rockville, MD, USA) in a 56 °C oven for 5 h and rinsed with 95% ethyl alcohol. The sections were differentiated in lithium carbonate solution (GeneCopoeia, Rockville, MD, USA) for 30 s and then in 70% ethyl alcohol until the white matter was sharply defined. The sections were counterstained in cresyl violet solution (GeneCopoeia, Rockville, MD, USA) for 30 s and differentiated in 95% ethyl alcohol for 5 min. The sections were dehydrated in 100% ethyl alcohol for 5 min twice, cleared with xylene twice for 5 min each time, and mounted with Permount medium. All sections were treated concomitantly for the same duration of time.

### TEM sample preparation and analysis

MOG-induced EAE mice and shiverer mice were anesthetized and perfused with normal saline followed by 2% paraformaldehyde/2.5% glutaraldehyde in 0.1 M phosphate buffer (PB, pH 7.4). The brains were excised and stored overnight at 4 °C in the same fixative. Small blocks of sectioned brains cut using a brain matrix were rinsed three times with PB for 10 min and then postfixed in 1% osmium tetroxide in 0.1 M PB for 90 min. The tissue was dehydrated in a series of graded ethanol solutions and embedded in an epoxy resin mixture. Ultrathin sections (70 nm) were cut using an ultramicrotome (UC7, Leica Microsystems, Wetzlar, Germany), mounted on 200-mesh grids, and contrasted with uranyl acetate and lead citrate. TEM images (15,000–60,000×) were randomly acquired by a TEM (H-7650, Hitachi, Tokyo, Japan) at an acceleration voltage of 80 kV.

### Statistical analysis

The data were analyzed by unpaired two-tailed Student’s *t* test and analysis of variance (ANOVA) using Prism 7 (GraphPad, San Diego, CA) software. The data are shown as the mean ± standard deviation (SD) for three to six replicates. *p* < 0.05 was considered statistically significant. *p < 0.05; **p < 0.01; ***p < 0.001; ****p < 0.0001.

### Reporting summary

Further information on research design is available in the Nature Research Reporting Summary linked to this article.

## Supplementary information

Supplementary Movie 1

Supplementary information

Reporting Summary

## Data Availability

All FASTQ files were uploaded to NCBI GEO database under accession codes GSE130063 and GSE130565.
