# Supplementary material for: OCT4-induced oligodendrocyte progenitor cells promote remyelination and ameliorate disease
Source: NPJ Regen Med. 2022 Jan 13;7:4. doi: 10.1038/s41536-021-00199-z (PMC8758684; doi:10.1038/s41536-021-00199-z)
Supplement: Supplementary file 2 — Supplementary information [file 41536_2021_199_MOESM2_ESM.pdf]

Supplementary information

Supplementary Figure 1

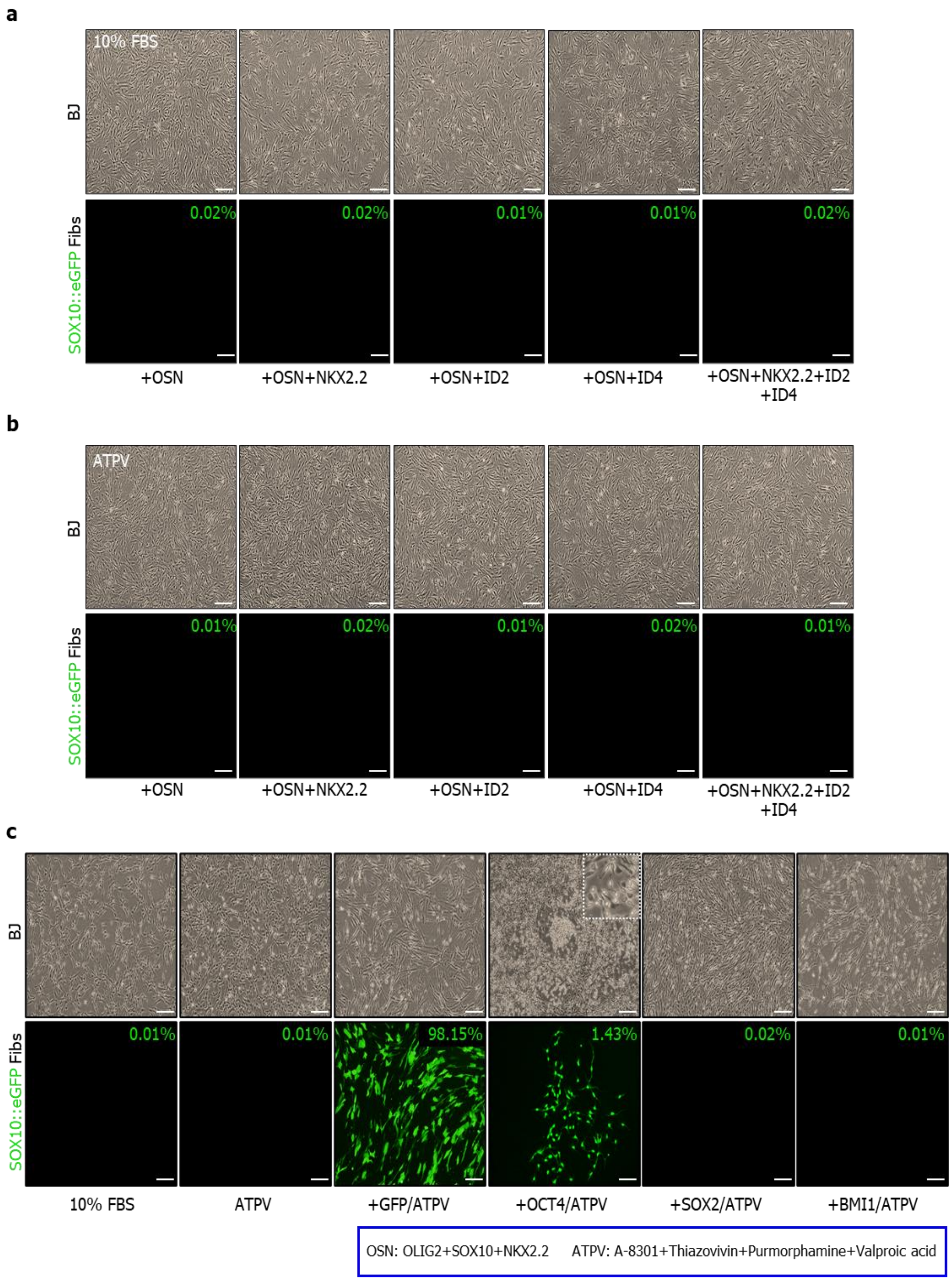

Supplementary Figure 1: Microscopy for GFP^+ cells in transcription factors-induced reporter cell line.

(**a, b**) Representative phase contrast (top, BJ) and green fluorescence images (bottom, SOX10::eGFP fibroblasts) on day 7 after transduction in combination with OSN (OLIG2, SOX10, and NKX6.2) or NKX2.2, ID2, and ID4. Transduced cells were cultured in medium containing 10% fetal bovine serum (FBS, **a**) or A-8301, thiazovivin, purmorphamine, and valproic acid (ATPV, **b**). Scale bars, 200 µm. (**c**) Representative images of human fibroblasts (BJ) on day 7 after transduction with pMXs-based retroviral vectors encoding GFP, OCT4, SOX2 or BMI1. Transduced cells were cultured in ATPV medium and non-transduced cells incubated with 10% FBS. Scale bars, 200 µm.

Supplementary Figure 2

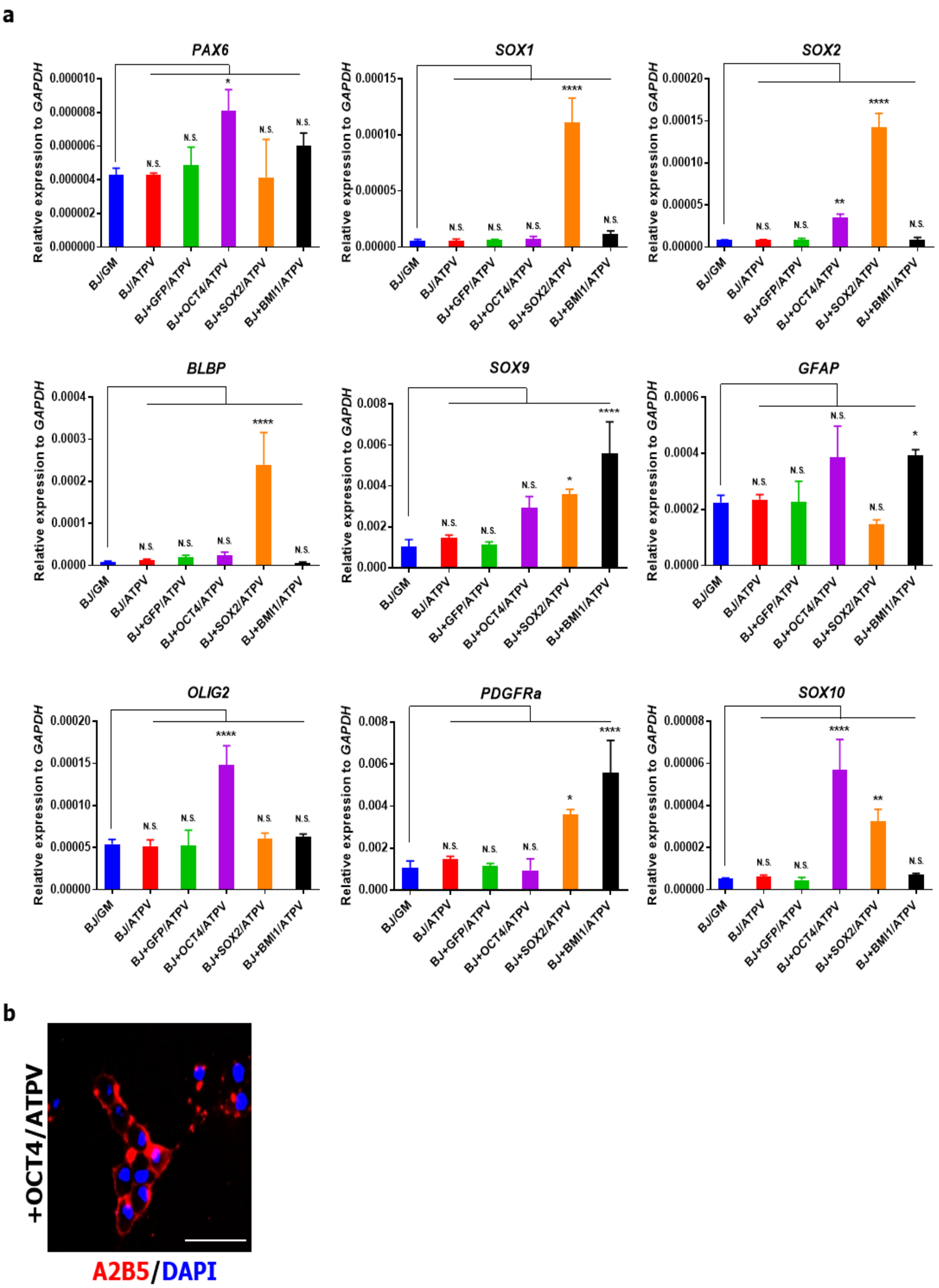

Supplementary Figure 2: Microscopy and qPCR analysis in transduced BJ.

(**a**) Comparative qPCR analyses were performed to identify the induced cells. Transduced BJ (human foreskin fibroblasts) were cultured in medium containing A-8301, thiazovivin, purmorphamine, and valproic acid (ATPV). Non-transduced cells incubated with 10% FBS (growth medium, GM) or ATPV. *PAX6*, *SOX1*, and *SOX2* represent early neuro-ectodermal markers; *BLBP*, *SOX9*, and *GFAP* represent astroglial markers; *OLIG2*, *SOX10*, and *PDGFRa* represent oligodendroglial markers. The transcript levels were normalized to *GAPDH*. Data are represented as the mean + SD (n=3). *, statistically significant difference vs. BJ/GM. Significant differences were analyzed by one-way ANOVA. N.S., not significant. (**b**) Immunofluorescence data showed that the induced cells expressed A2B5, which is a typical OPC marker. Scale bars, 100 µm.

Supplementary Figure 3

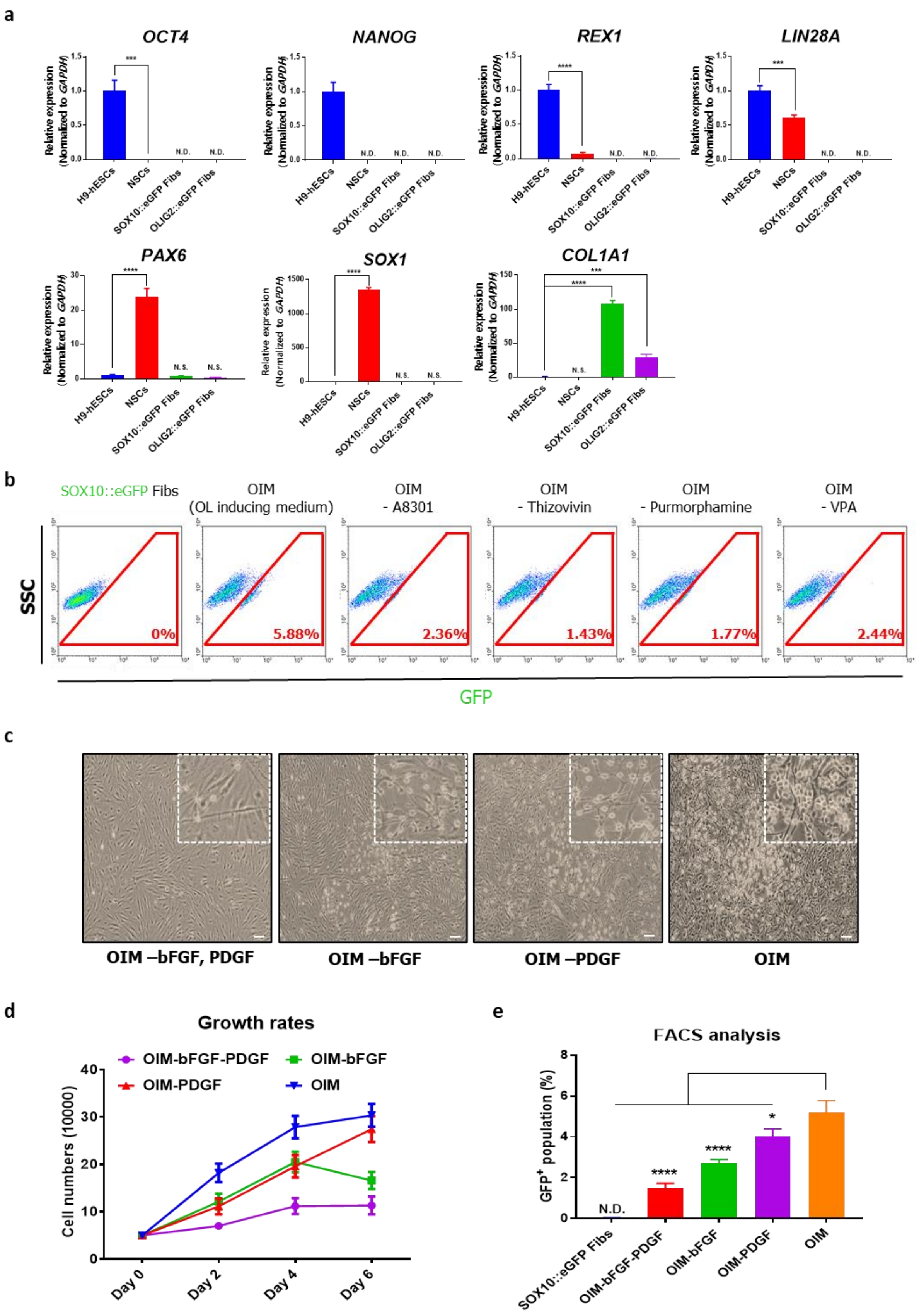

Supplementary Figure 3: qPCR analysis and optimization of OPC-inducing culture conditions.

(**a**) qPCR analysis in H9-hESCs, NSCs, and OLIG2::eGFP fibroblasts, and SOX10::eGFP fibroblasts. NSCs were differentiated from H9-hESCs. The expression levels are shown relative to those of H9-hESCs, and the transcript levels were normalized to *GAPDH*. The data are represented as the mean + SD (n=3). *, statistically significant difference vs. H9-hESCs. Significant differences were analyzed by one-way ANOVA. N.S., not significant; N.D., not detected. (**b**) The effect of the withdrawal of each small molecule from the ATVP cocktail on the $SOX10^+$ population from a sample of OCT4-induced SOX10::eGFP fibroblasts cultured in the presence of forskolin (OIM). The data from three independent flow cytometry experiments are presented as the mean values. (**c**) Representative images of OCT4-induced SOX10::eGFP fibroblasts cultured in OIM with or without bFGF and PDGF-AA until induction day 6. Scale bars, 200 μm. (**d**) Cell proliferation curves for the populations of reprogrammed cells cultured in OIM with or without bFGF and PDGF-AA until induction day 6. (**e**) The effect of the withdrawal of each cytokine on the $SOX10^+$ population from a sample of OCT4-induced SOX10::eGFP fibroblasts. The data from three independent flow cytometry experiments are presented as the mean values. *, statistically significant difference vs. OIM. Significant differences were analyzed by one-way ANOVA. N.D., not detected.

Supplementary Figure 4

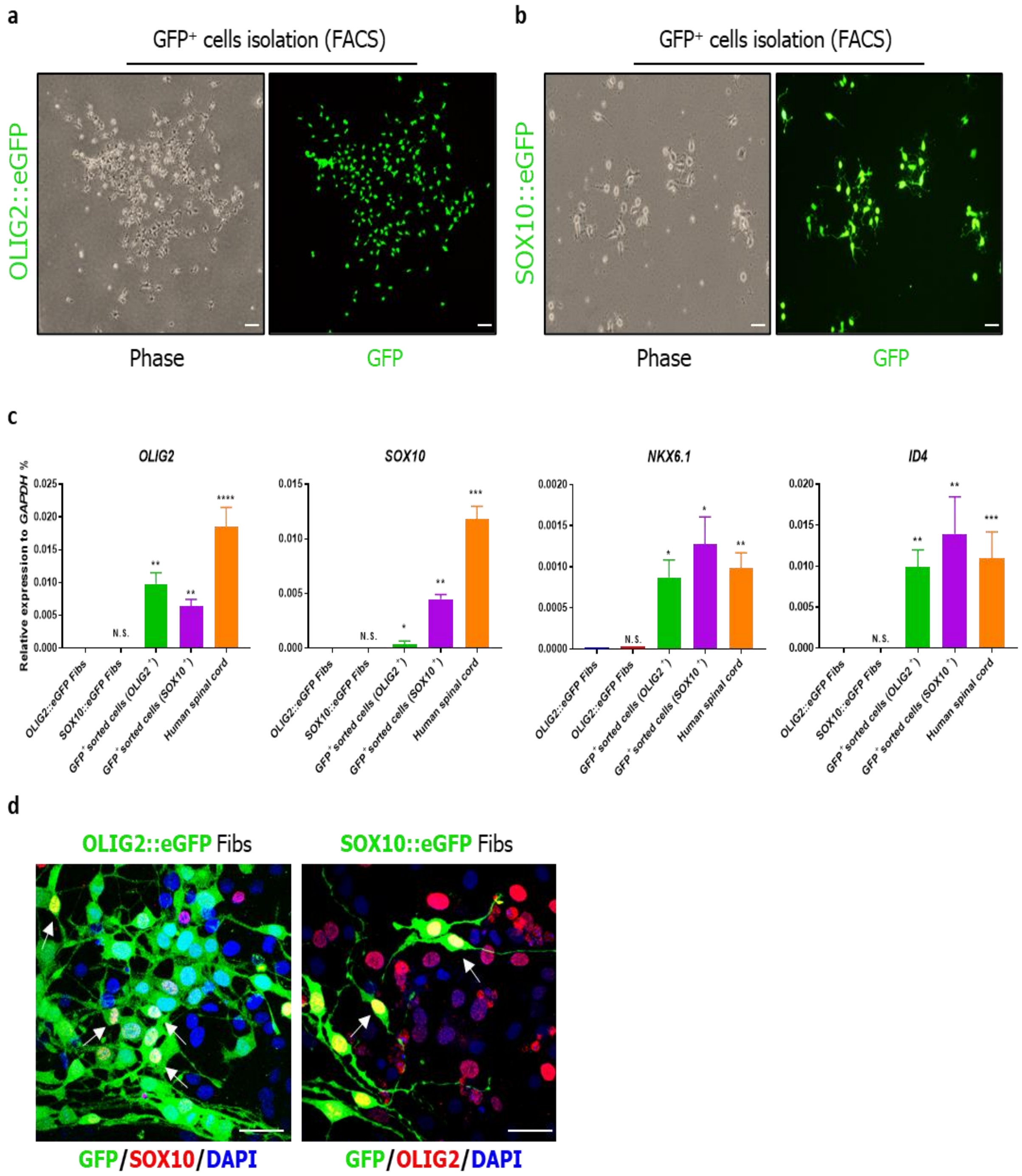

Supplementary Figure 4: Microscopy and qPCR analysis for OPC marker in GFP⁺ cells derived from OCT4-induced reporter cell lines.

GFP-positive cells sorted from OCT4-induced OLIG2::eGFP and SOX10::eGFP fibroblasts after 14 days of induction were subjected to the following analyses. (**a, b**) Representative phase contrast (left) and green fluorescence images (right) of GFP-sorted cells from a sample of OCT4-induced OLIG2::eGFP and SOX10::eGFP fibroblasts by FACS. Scale bars, 100 µm. (**c**) Comparative qPCR analysis for OPC markers in GFP-positive cells sorted from OCT4-induced OLIG2::eGFP fibroblasts and SOX10::eGFP fibroblasts. The expression levels are shown relative to those of OLIG2::eGFP fibroblasts and the transcript levels were normalized to *GAPDH*. The RNA obtained from human spinal cord served as control. The data are represented as the mean + SD (n=3). *, statistically significant difference vs. OLIG2::eGFP fibroblasts. *P < 0.05, **P < 0.01, ***P < 0.001, ****P < 0.0001 by one-way ANOVA (N.S., not significant). (**d**) Representative immunofluorescence images for the coexpression of OLIG2 and SOX10 in OCT4-transduced reporter cell lines, cultured in OIM for 10 days. Arrows denote representative coexpressing cells. Scale bars, 100 µm.

Supplementary Figure 5

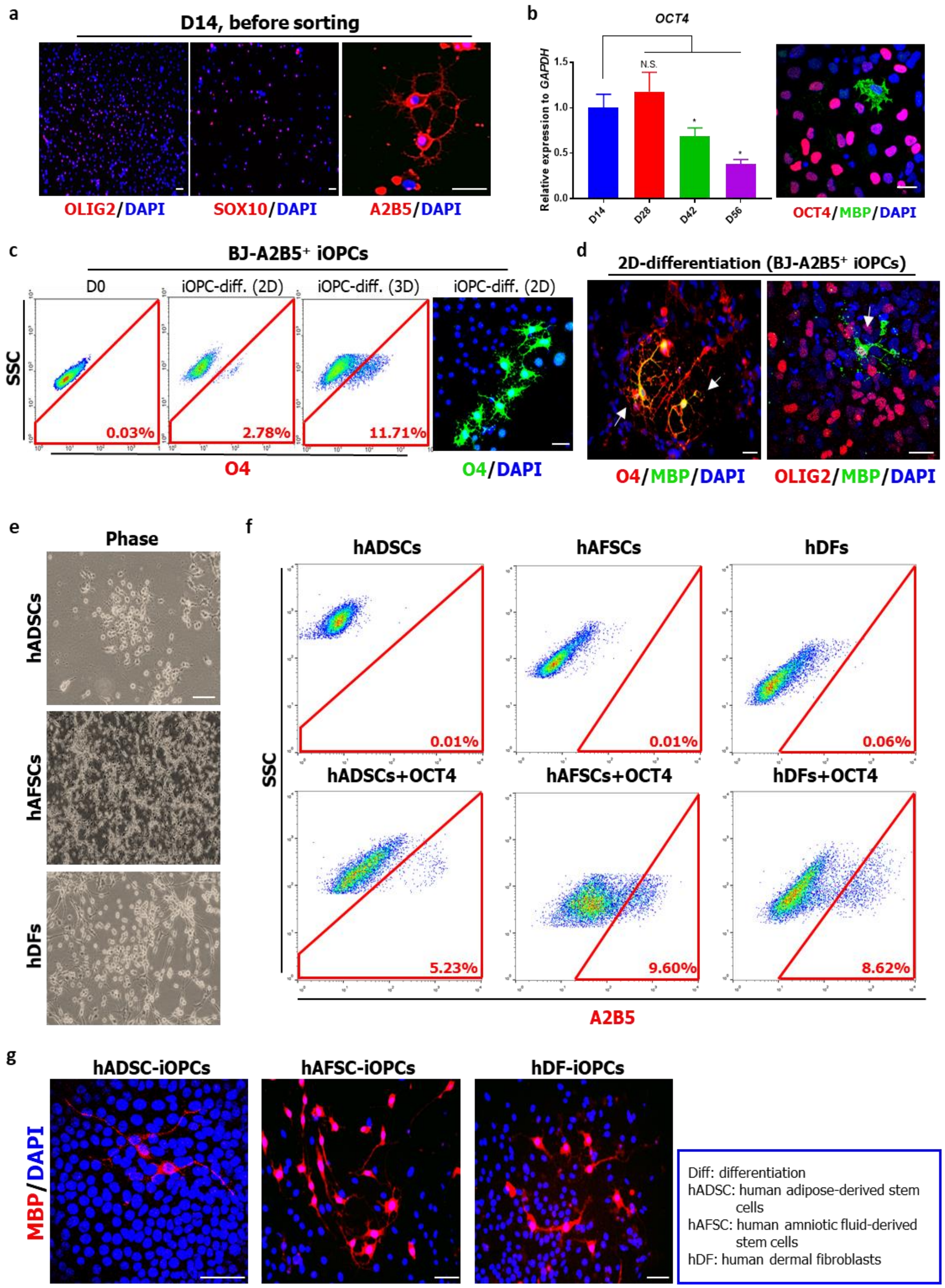

Supplementary Figure 5: Characterization of A2B5$^+$ iOPCs derived from human fibroblasts.

(**a**) Representative immunofluorescence images show that reprogrammed BJ cells express OLIG2, SOX10, and A2B5 on day 14 after OCT4 transduction. Scale bars, 100 µm. (**b**) Comparative qPCR analysis (left) for expression of *OCT4* during differentiation in iOPCs. The expression levels of *OCT4* at different stages of differentiation are shown relative to those of day 14, and the transcript levels were normalized against *GAPDH*. The data are represented as the mean + SD (n=3). *, statistically significant difference vs. D14. *$P < 0.05$ by one-way ANOVA (N.S., not significant). Representative fluorescence images (right) for the differentiation (2D) of A2B5$^+$ iOPCs into oligodendrocytes. Scale bars, 100 µm. (**c**) Representative flow cytometry data for O4$^+$ cells differentiated from A2B5$^+$ iOPCs using 2D (monolayer) and 3D (sphere) culture. The data from three independent experiments are presented as the mean values. O4$^+$ cells were further validated by immunocytochemistry (right). Scale bars, 100 µm. (**d**) Representative fluorescence images of the differentiation (2D) of A2B5$^+$ iOPCs into oligodendrocytes at day 50. The arrows denote coexpressed cells. Scale bars, 100 µm. (**e**) Representative images of reprogrammed human ADSCs, AFSCs, and DFs at day 10. Scale bars, 100 µm. (**f**) Representative flow cytometry data for A2B5$^+$ cell populations at day 14 among cells reprogrammed with OCT4. The data from three independent experiments are presented as the mean values. (**g**) Representative fluorescence images for the differentiation (2D) of several human A2B5$^+$ iOPCs into oligodendrocytes. The expression of MBP was observed in differentiated cells. Scale bars, 100 µm.

Supplementary Figure 6

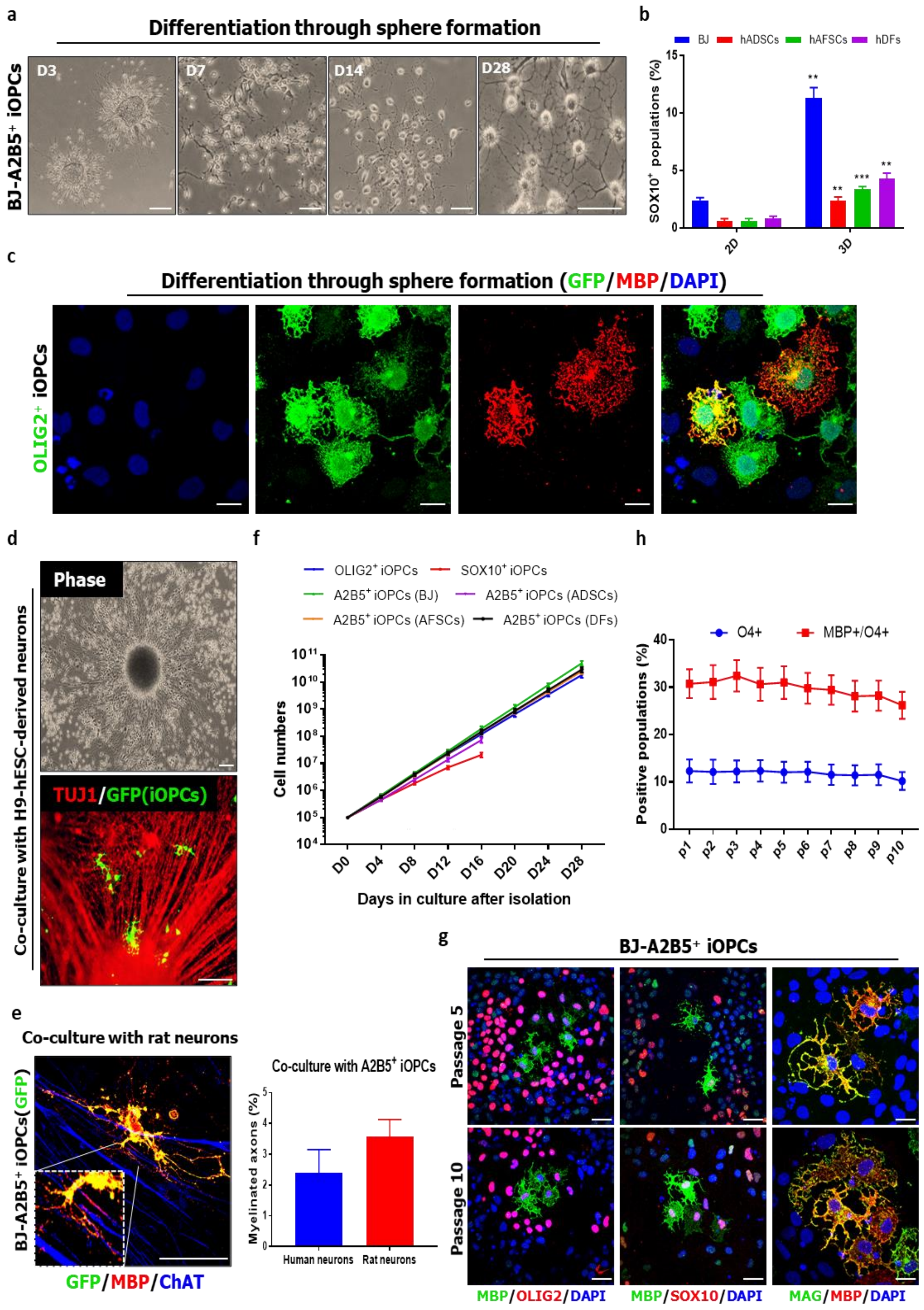

Supplementary Figure 6: 3D-differentiation of A2B5$^+$ iOPCs derived from human fibroblasts.

(**a**) Representative phase-contrast microscopy images of sphere-derived cells (A2B5$^+$ iOPCs) on days 3, 7, 14, and 20 after seeding on PLO/laminin-coated dishes. Scale bars, 100 µm. (**b**) Comparative quantitative analysis of SOX10$^+$ populations resulting from differentiation (2D or 3D) of A2B5$^+$ iOPCs. Data are represented as means ± SD (n=3). (**c**) Representative fluorescence images of the differentiation (3D) of OLIG2$^+$ iOPCs (derived from OLIG2::eGFP fibroblasts) into oligodendrocytes in sphere culture. The expression of MBP was observed in the GFP$^+$ cells. Scale bars, 100 µm. (**d, e**) GFP-tagged A2B5$^+$ iOPCs (BJ) were cocultured with H9-hESC-derived neurons (D) and rat spinal cord-derived neurons (E). Magnified pictures (3X) of the boxed areas in the corresponding images were shown on the image. Scale bars, 200 µm. (**f**) Growth curves demonstrating proliferation of OLIG2$^+$ iOPCs, SOX10$^+$ iOPCs, A2B5$^+$ iOPCs (BJ), A2B5$^+$ iOPCs (ADSCs), A2B5$^+$ iOPCs (AFSCs), and A2B5$^+$ iOPCs (DFs). Each point refers to the cell numbers of various iOPCs. (**g**) Representative fluorescence images of the differentiation (3D) of BJ-A2B5$^+$ iOPCs (Passage 5 and 10) into oligodendrocytes in sphere culture. The expression of MBP with the coexpression OLIG2, SOX10 and MAG was observed. Scale bars, 100 µm. (**h**) Quantitative analysis of O4$^+$ and MBP$^+$/O4$^+$ populations resulting from differentiation (3D) of BJ-A2B5$^+$ iOPCs at different passages. Data are represented as means ± SD (n=3).

Supplementary Figure 7

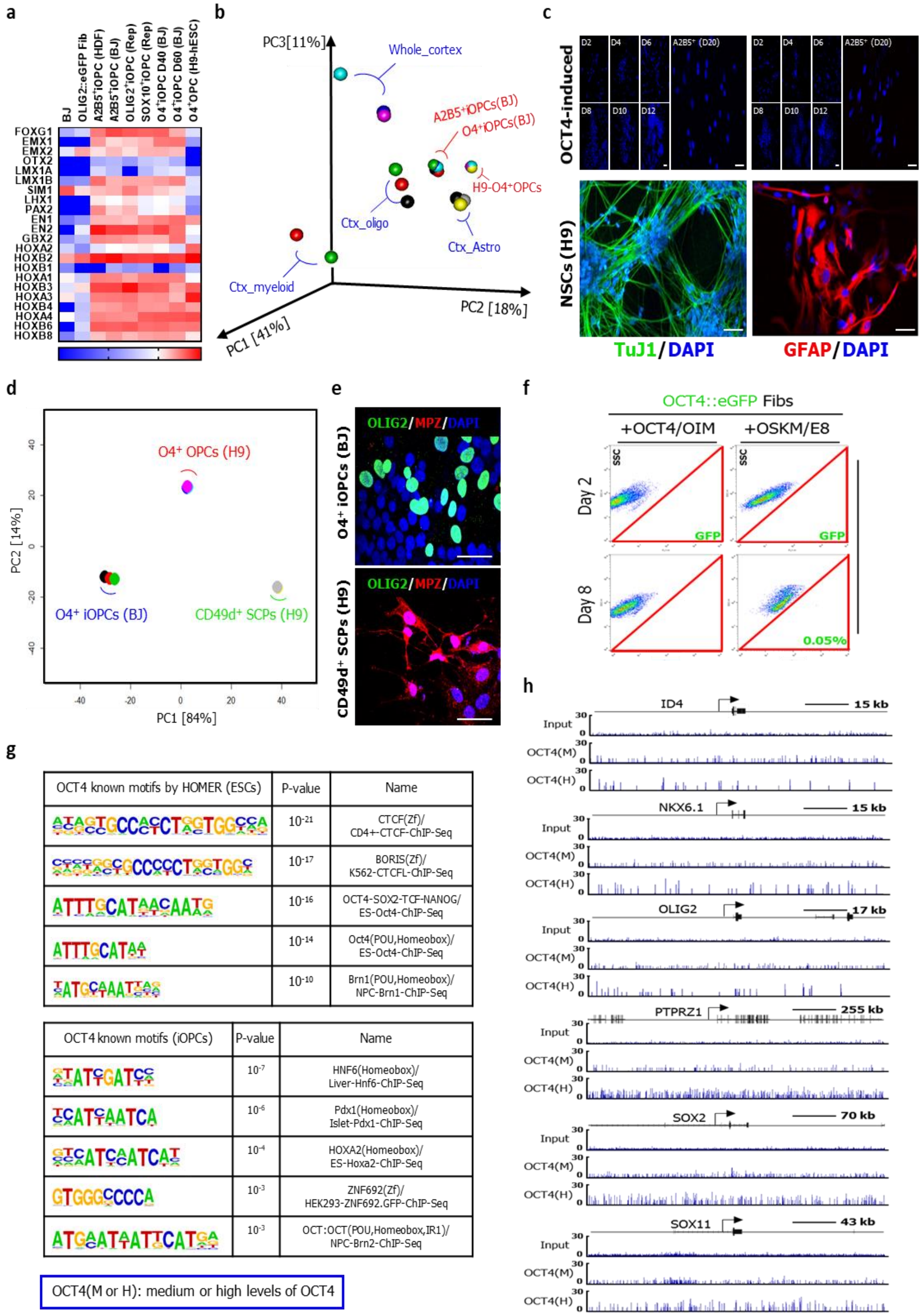

Supplementary Figure 7: Detailed profiles of various iOPCs and analysis of genome-wide sequencing upon OCT4-induced reprogramming.

(**a**) A heatmap of region-specific markers identified in RNA-seq profiles. Red: upregulated genes; Blue: downregulated genes. (**b**) Principal-component analysis (PCA) with human brain-derived cells produced in previous study (Zhang *et al*, 2016). Ctx_myeloid, cortex-derived myeloid cells; Ctx_Oligo, cortex-derived oligodendrocytes; Ctx_astro, cortex-derived astrocytes. (**c**) The *in vitro* differentiation of NSCs and iOPCs into neurons ($TuJ1^+$) and astrocytes ($GFAP^+$). Scale bars, 100 µm. (**d**) PCA of $O4^+$ iOPCs (BJ), $O4^+$ OPCs (H9), and $CD49d^+$ SCPs (H9). RNA-seq expression profile of $CD49d^+$ SCPs were obtained in previous study. (**e**) The *in vitro* differentiation of iOPCs and SCPs into MPZ^+ myelinating cells. Scale bars, 50 µm. (**f**) Representative flow cytometry data for the proportion of GFP^+ cells in OCT4::eGFP fibroblasts transduced with OCT4 cultured in OIM and transduced with OSKM cultured in E8 medium. The data from three independent experiments are presented as the mean values. (**g**) The transcription factor-binding motifs targeted by OCT4 in H9-hESCs (top) and $A2B5^+$ iOPCs ($iOPCs^{high}$, bottom) as analyzed by HOMER software. (**h**) Genomic snapshots of the ChIP-seq signal of OCT4 as representative examples of putative OCT4 targets in OPC development.

Supplementary Figure 8

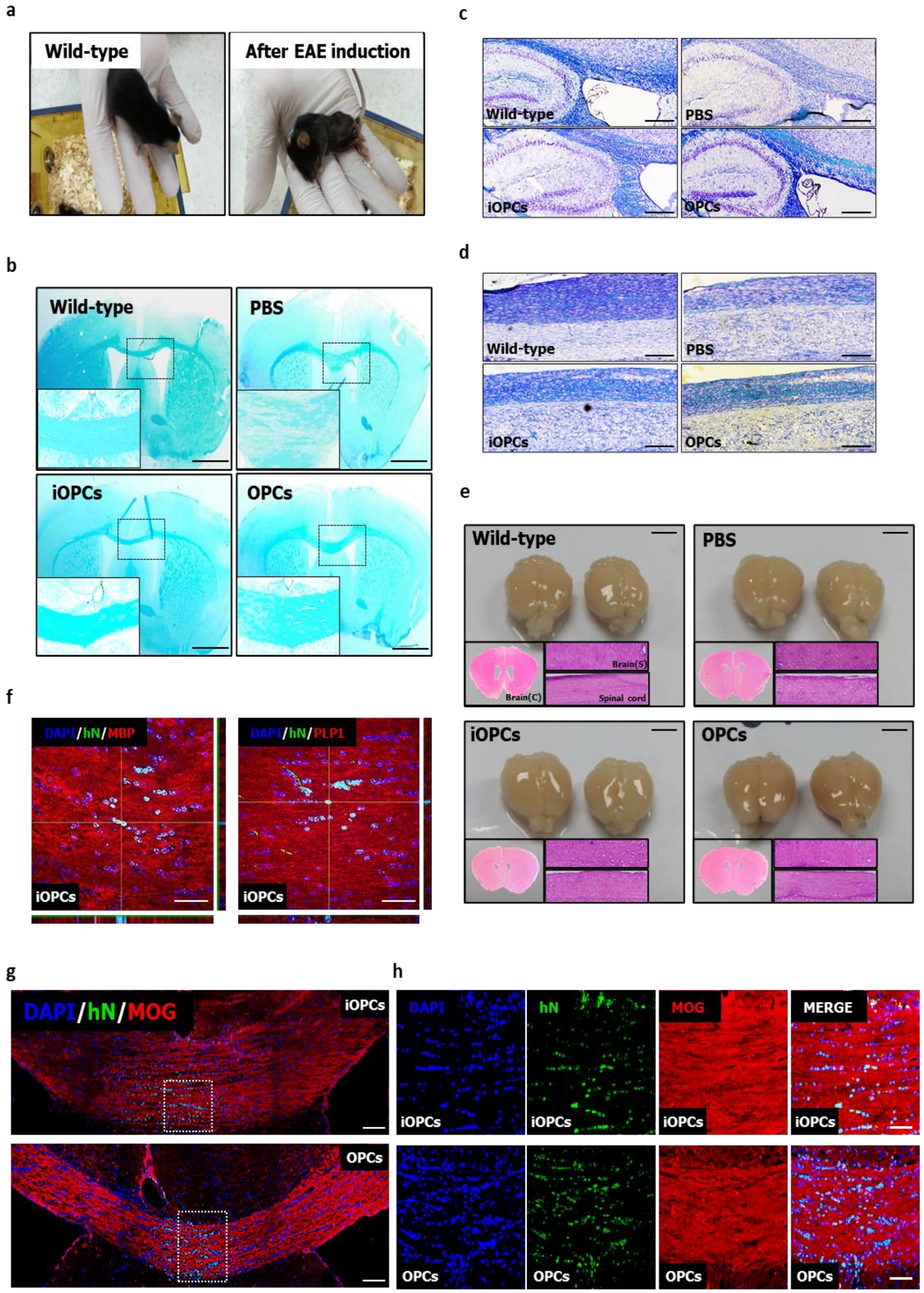

Supplementary Figure 8: Analysis of remyelination and disease amelioration upon transplantation of iOPCs in mouse EAE model.

(**a**) MOG-induced mice exhibited motor dysfunction and paralysis which represent successful induction of EAE onset. EAE mice were randomly divided into three groups (PBS, iOPCs, and OPCs) before transplantation. (**b-d**) Luxol fast blue staining in the corpus callosum of the brains (**b**, coronal view; **c**, sagittal view) and spinal cord (**d**, sagittal view) of EAE-induced mice. LFB staining patterns showed that iOPC-engrafted brains have abundant myelin comparable to normal mice, whereas PBS-treated mice exhibited poor myelin staining. Scale bars, 1 mm (**b**) and 200 μm (**c**, **d**). (**e**) Representative photographs of normal and EAE-induced mouse brains. There were no signs of tumors in brain of transplanted mice. H&E-stained brains and spinal cords of the transplanted mice exhibited normal histopathological structures, similar to wild-type mice. Scale bars, 5 mm. (**f**) Representative confocal images illustrating the *in vivo* differentiation of iOPCs into MBP^+ oligodendrocytes (left) and $PLP1^+$ oligodendrocytes (right). The transplanted iOPCs were identified using a human-specific nuclear antibody. Scale bars, 40 μm. (**g**) The transplantation of iOPCs and OPCs into the corpus callosum of EAE-induced mice resulted in an abundance of MOG-positive populations. The transplanted iOPCs and OPCs were identified by human specific nuclear antigens. Scale bars, 100 μm. (**h**) Magnified pictures of the boxed areas in the corresponding images in (**g**). Scale bars, 50 μm.

Supplementary Figure 9

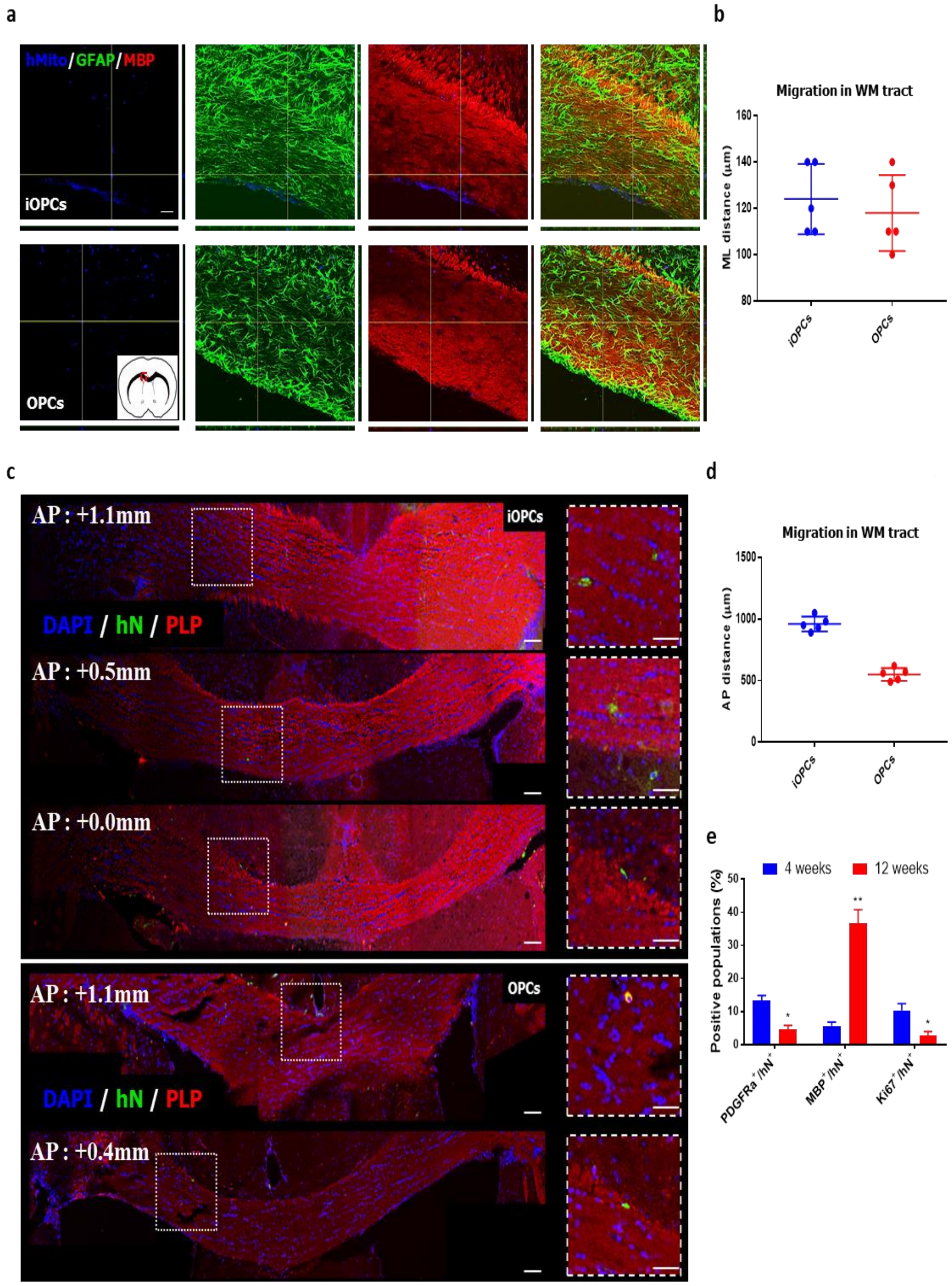

Supplementary Figure 9: Confocal microscopy analysis of transplanted iOPCs and their evaluation of *in vivo* migration (medial-lateral and anterior-posterior) in the EAE model.

(**a, b**) Representative confocal images illustrating the *in vivo* migration (medial-lateral) of iOPCs and OPCs (**a**). The transplanted iOPCs and OPCs were identified using a human-specific anti-mitochondrial antibody and the evaluation of the distance of dispersal of engrafted cells in serial coronal sections (**b**). Scale bars, 50 μm. (**c, d**) Representative confocal images illustrating the *in vivo* migration (anterior-posterior) of iOPCs and OPCs (**c**). The right panels represent magnified pictures of the boxed areas in the corresponding left panels. The transplanted iOPCs were identified using human specific nuclear antigens and the evaluation of the distance of dispersal of engrafted cells in serial coronal sections (**d**). Scale bars, 100 μm. (**e**) The percentage of OPCs and oligodendrocytes at week 4 and 12 post-grafted.

Supplementary Tables

Supplementary Table 1. Small molecules used in the present study.

Name	Target	Effect	Working concentration
EX-527	SIRT1 inhibitor	Promotes oligodendrocyte differentiation in NSCs	5 μM
(±) BayK 8644	Calcium channel agonist	Promotes neural differentiation and iPSC generation	2 μM
RG-108	DNA methyltransferase inhibitor	Improve the reprogramming efficiency and promote reprogramming mouse fibroblasts to NSCs	0.5 μM
DMH1	selective ALK2 inhibitor	Promotes neural differentiation in hESCs and hiPSCs	1 μM
Retinoic acid (All-trans)	Ligand for both RAR and RXR	Promotes neural and oligodendrocyte differentiation in hESCs and hiPSCs	0.1 μM
Forskolin	cAMP/PKA signaling agonist.	Promotes oligodendrocytes differentiation in NSCs and reprogramming	10 μM
lysophosphatidic acid	Ligand for LPA receptor signaling	Inhibits neural differentiation in NSCs	2 μM
Parnate	LSD1 inhibitor	Improve reprogramming efficiency	2 μM
Dexamethasone	Glucocorticoid signaling agonist	Promotes oligodendrocyte differentiation in NSCs	1 μM
CHIR99021	GSK-3 inhibitor	Enhances ESCs/iPSCs self–renewal and enables iPSC generation	3 μM

Supplementary Table 2. Summary of positive populations for each marker.

Cell type (iOPCs)	Mean ± SD%					
	$OLIG2^+$ (D14)	$SOX10^+$ (D14)	$A2B5^+$ (D14)	$A2B5^+OLIG2^+$ (D14)	$A2B5^+SOX10^+$ (D14)	$O4^+$ (D40)
OLIG2::eGFP Fibroblasts	18.49 ± 2.81%	4.58 ± 1.12%	12.36 ± 2.84%	11.06 ± 2.24%	1.34 ± 0.36%	7.46 ± 2.23%
SOX10::eGFP Fibroblasts	14.62 ± 3.22%	5.25 ± 1.71%	11.40 ± 2.72%	9.92 ± 2.24%	1.82 ± 0.54%	13.64 ± 4.63%
BJ	17.56 ± 3.61%	3.80 ± 0.89%	15.37 ± 3.48%	14.03 ± 3.19%	1.44 ± 0.50%	11.71 ± 2.25%
hADSCs	6.67 ± 1.34%	1.37 ± 0.35%	5.23 ± 1.51%	4.66 ± 1.28%	0.61 ± 0.23%	2.09 ± 0.67%
hAFSCs	10.68 ± 2.80%	2.09 ± 0.52%	9.60 ± 2.73%	8.46 ± 2.44%	0.65 ± 0.20%	3.43 ± 1.78%
hDFs	11.49 ± 2.34%	2.83 ± 0.42%	8.62 ± 2.32%	7.53 ± 1.99%	0.86 ± 0.23%	3.21 ± 0.91%

Supplementary Table 3. Summary of EAE induced animal study.

Groups	Mean disease scores (after engraftment, mean ± SEM)				
	Day 1	Day 5	Day 10	Day 50	Day 100
PBS (n=6)	3.5±0.5	3.0±0.16	3.0±0.2	3.0±0.19	3.08±0.20
$O4^+$ iOPCs (n=8)	3.33±0.57	2.08±0.28	1.58±0.28	1.42±0.3	1.58±0.36
$O4^+$ OPCs (n=8)	3.16±0.48	2.08±0.24	1.5±0.35	1.33±0.34	1.44±0.32

Supplementary Table 4. Sequences of the primers used for RT-PCR and qPCR.

Genes	Primer sequences (5' to 3')	Type
OCT4	GACAGGGGGAGGGGAGGAGCTAGG	Forward
	CTTCCCTCCAACCAGTTGCCCCAAAC	Reverse
SOX2	TCAGGAGTTGTCAAGGCAGAGA	Forward
	CCGCCGCCGATGATTGTTATTA	Reverse
REX1	CTGAAGAAACGGGCAAAGAC	Forward
	GAACATTCAAGGGAGCTTGC	Reverse
NANOG	CAGCCCTGATTCTTCCACCAGTCCC	Forward
	GGAAGGTTCCCAGTCGGGTTCACC	Reverse
LIN28	GCTCCGTGTCCAACCAGCAG	Forward
	TTTCCTTTTGGCCGCCTCTC	Reverse
PAX6	GGCTCAAATGCGACTTCAG	Forward
	CCCTTCGATTAGAAAACCATACC	Reverse
SOX1	ACACTTGAAGCCCAGATGG	Forward
	ATAGGCTCACTTTTGGACGG	Reverse
FABP7	GATTTTTGCCCACCCTCTTTCCAA	Forward
	CGCCTAGAGCCTTCATGTACTCAT	Reverse
SOX9	GGCAACTCGTACCCAAATTTCCAA	Forward
	CGCCTAGAGCCTTCATGTACTCAT	Reverse
GFAP	GGCAACTCGTACCCAAATTTCCAA	Forward
	TCACACGATTCTCCATCATCCTCC	Reverse
OLIG2	AGCGCTGATGGTCATATCCAATCT	Forward
	CCTGAAATCGGCAGTTTTGGGTTA	Reverse
PDGFRα	CAGTCCTGGTGCTGTTGGTG	Forward
	CCGGCTTAATCCATAGGCTG	Reverse
S100β	GGAGACAAGCACAAGCTGAA	Forward
	CTTGCATGACCGTCTCTGTT	Reverse
SOX10	AAGCCCAGGTGAAGACAGAGAC	Forward
	CCATATAGGAGAAGGCCGAGTAGA	Reverse
ZFP536	AAGCAGTTTGGTGTTTACCCAGG	Forward
	GGAGGCAATGTCAGAGAGGTCAT	Reverse
NKX2.2	GCTTTTCGTTGTAAATATCGGTGG	Forward
	GCCTACAGGGTTTTCTTTTCCATA	Reverse
PLP1	GCTCTGCTGTGCCTGTGTACATT	Forward
	ATCATGAAGGTGAGCAGGGAAAC	Reverse
MBP	CCAGAGCAGCCTCTATGAACAA	Forward
	GGAAGTGAATGAGCCGGTTATC	Reverse
GAPDH	GTGGTCTCCTCTGACTTCAACA	Forward
	CTCTTCCTCTTGTGCTCTTGCT	Reverse
SOX10 promoter (bisulfite)	TGGGTAAGGTTAAGAAGGAGTAGTAG	Forward
	CTACCTAAACCCACACCATAAAAAC	Reverse

Supplementary Table 5. List of Primary Antibodies.

Primary antibody	Dilution	Host	Provider
OCT4	1:200	Mouse	Santa cruz (SC-5279)
OCT4 (ChIP)	3 µg	Mouse	Santa cruz (SC-5279X)
OLIG2	1:200	Rabbit	Merck Millipore (AB9610)
NKX2.2	1:100	Mouse	DSHB (74.5A5)
A2B5	1:200	Mouse	R&D Systems (MAB1416)
PDGFRα	1:200	Rabbit	Santa cruz (SC-338)
SOX10	1:100	Goat	R&D Systems (AF2864)
S100β	1:500	Mouse	Sigma-Aldrich (S2532)
GFAP	1:500	Rabbit	Merck Millipore (AB5804)
O4	1:200	Mouse	R&D Systems (MAB1326)
O4	1:50	Mouse	Merck Millipore (MAB345)
MOG	1:100	Goat	Abcam (AB115597)
MOG	1:100	Mouse	Merck Millipore (MAB5680)
MBP	1:50	Rat	Abcam (AB7349)
MBP	1:100	Rat	Merck Millipore (MAB386)
PLP1	1:200	Rabbit	Abcam (AB28486)
TuJ1	1:1000	Mouse	BioLegend (801202)
hNuclei (HuNu)	1:100	Mouse	Merck Millipore (MAB1281)
hMitochondria	1:100	Mouse	Merck Millipore (MAB1273)
Neurofilament 200	1:200	Rabbit	Sigma-Aldrich (N4142)
MPZ	1:200	Rat	Novus Biologicals (NB100-1607)
GFP	1:200	Rabbit	Abcam (ab6556)
GFP	1:200	Mouse	Abcam (ab1218)

Supplementary Table 6. List of Secondary Antibodies.

Secondary antibody	Dilution	Host	Provider
Alexa Fluor 488 (Mouse IgG)	1:500	Donkey	Thermo Fisher (A-21202)
Alexa Fluor 488 (Rabbit IgG)	1:500	Donkey	Thermo Fisher (A-21206)
Alexa Fluor 488 (Goat IgG)	1:500	Donkey	Thermo Fisher (A-11055)
Alexa Fluor 488 (Mouse IgM)	1:500	Goat	Thermo Fisher (A-21042)
Alexa Fluor 488 (Rat IgG)	1:500	Donkey	Thermo Fisher (A-21208)
Alexa Fluor 594 (Mouse IgG)	1:500	Donkey	Thermo Fisher (A-21203)
Alexa Fluor 594 (Rabbit IgG)	1:500	Donkey	Thermo Fisher (A-21207)
Alexa Fluor 594 (Goat IgG)	1:500	Donkey	Thermo Fisher (A-11058)
Alexa Fluor 594 (Rat IgG)	1:500	Donkey	Thermo Fisher (A-21209)
Alexa Fluor 647 (Mouse IgG)	1:500	Donkey	Thermo Fisher (A-31571)
Alexa Fluor 647 (Rabbit IgG)	1:500	Donkey	Thermo Fisher (A-31573)
Alexa Fluor 647 (Goat IgG)	1:500	Donkey	Thermo Fisher (A-21447)

Supplementary Movie legend

Supplementary Movie 1. **Behavioral investigation of mice with experimental autoimmune (Related to Fig. 6).**

Eleven-week-old C57BL/6 mice were immunized subcutaneously with an emulsion of MOG_{35-55} in complete Freund's adjuvant (CFA) and were then administered pertussis toxin (PTX) (Hooke Kit TM MOG_{35-55}/CFA Emulsion PTX) on the day of immunization and then again the following day. Once the mice reached the peak of disease (~day 14; clinical score=3.5), they were randomized and received PBS or iOPCs.

(A) The representative MOG_{35-55} induced experimental autoimmune encephalomyelitis (EAE) mice on day 14 after immunization.

(B) The representative PBS-treated MOG_{35-55} induced EAE mice on day 100 after surgery.

(C) The representative iOPCs-engrafted MOG_{35-55} induced EAE mice on day 100 after surgery.

(D) The representative 27-week-old wild-type C57BL/6 mice on day 100 after surgery (nonimmunized control mice).
