# Supplementary material for: OCT4-induced oligodendrocyte progenitor cells promote remyelination and ameliorate disease
Source: NPJ Regen Med. 2022 Jan 13;7:4. doi: 10.1038/s41536-021-00199-z (PMC8758684; doi:10.1038/s41536-021-00199-z)
Supplement: Supplementary file 3 — Reporting Summary [file 41536_2021_199_MOESM3_ESM.pdf]

nature portfolio

Corresponding author(s): Seungkwon You

Last updated by author(s): Sep 6, 2021

Reporting Summary

Nature Portfolio wishes to improve the reproducibility of the work that we publish. This form provides structure for consistency and transparency in reporting. For further information on Nature Portfolio policies, see our Editorial Policies and the Editorial Policy Checklist.

Statistics

For all statistical analyses, confirm that the following items are present in the figure legend, table legend, main text, or Methods section.

n/a	Confirmed	
☐	☒	The exact sample size (*n*) for each experimental group/condition, given as a discrete number and unit of measurement
☐	☒	A statement on whether measurements were taken from distinct samples or whether the same sample was measured repeatedly
☐	☒	The statistical test(s) used AND whether they are one- or two-sided<br>*Only common tests should be described solely by name; describe more complex techniques in the Methods section.*
☒	☐	A description of all covariates tested
☐	☒	A description of any assumptions or corrections, such as tests of normality and adjustment for multiple comparisons
☐	☒	A full description of the statistical parameters including central tendency (e.g. means) or other basic estimates (e.g. regression coefficient) AND variation (e.g. standard deviation) or associated estimates of uncertainty (e.g. confidence intervals)
☐	☒	For null hypothesis testing, the test statistic (e.g. *F*, *t*, *r*) with confidence intervals, effect sizes, degrees of freedom and *P* value noted<br>*Give P values as exact values whenever suitable.*
☒	☐	For Bayesian analysis, information on the choice of priors and Markov chain Monte Carlo settings
☒	☐	For hierarchical and complex designs, identification of the appropriate level for tests and full reporting of outcomes
☒	☐	Estimates of effect sizes (e.g. Cohen's *d*, Pearson's *r*), indicating how they were calculated

Our web collection on statistics for biologists contains articles on many of the points above.

Software and code

Policy information about availability of computer code

Data collection

no software was used.

Data analysis

RNA-seq analysis was performed as described in (http://master.bioconductor.org/packages/release/workflows/html/rnaseqGene). The reads were aligned to hg19 using Bowtie2, gene expression levels were measured with Cufflinks v2.1.1 using Ensembl gene annotation database and analyzed by DESeq2.
For ChIP-seq analysis, the reads were aligned to hg19 using Bowtie2; MACS and HOMER were used to identify peaks and motif from alignment file. The IGV browser was used to visualize peak locations.

GSEA was performed following the workflow: https://software.broadinstitute.org/gsea/doc/GSEAUserGuideFrame

ImageJ was used to analyze G-ratio

Flowjo software was used to get quantified images from flow-cytometry analysis

For manuscripts utilizing custom algorithms or software that are central to the research but not yet described in published literature, software must be made available to editors and reviewers. We strongly encourage code deposition in a community repository (e.g. GitHub). See the Nature Portfolio guidelines for submitting code & software for further information.

Data

Policy information about availability of data

All manuscripts must include a data availability statement. This statement should provide the following information, where applicable:

- Accession codes, unique identifiers, or web links for publicly available datasets
- A description of any restrictions on data availability
- For clinical datasets or third party data, please ensure that the statement adheres to our policy

All Sequencing data generated in present study were deposited at NCBI GEO (RNA-seq, GSE130063; ChIP-seq, GSE130565).

Field-specific reporting

Please select the one below that is the best fit for your research. If you are not sure, read the appropriate sections before making your selection.

☒ Life sciences ☐ Behavioural & social sciences ☐ Ecological, evolutionary & environmental sciences

For a reference copy of the document with all sections, see nature.com/documents/nr-reporting-summary-flat.pdf

Life sciences study design

All studies must disclose on these points even when the disclosure is negative.

Sample size	We conducted a minimum of three independent biological replicates for whole experiments including viral production (more than n=30), OPC induction with chemical treatment (more than n=30), PCR, flow-cytometry, deep-sequencing and animal study (In vivo, more than n=6 per group)
Data exclusions	In reprogramming study, viruses exhibiting poor transduction efficiency (<90%) were excluded and OCT4-transduced fibroblasts exhibiting poor viability and expressing low levels of OCT4 (transgene) were excluded. In the behavior study of EAE-induced mice, we excluded three mice from PBS-engrafted group, two mice from iOPC-engrafted group, and three mice from OPC-engrafted group which could not survive until 100 days
Replication	Our methods for OCT4-induced experiments were repeated through a minimum of 20 biological replicates in human foreskin fibroblasts (BJ) and also were reproducible in 5 types of different human fibroblasts through a minimum of 3 biological replicates
Randomization	For in vitro analysis, additional three members of our laboratory and one member of other laboratory (Prof. Hong's) successfully generated iOPCs using this methods and for in vivo, before engraftment, the EAE-induced mice randomized into three group (PBS-treated, iOPCs-engrafted, and OPCs-engrafted).
Blinding	In behavior analysis, the experimenters who assessed and scored the mice daily were blinded to the identity of the animals

Reporting for specific materials, systems and methods

We require information from authors about some types of materials, experimental systems and methods used in many studies. Here, indicate whether each material, system or method listed is relevant to your study. If you are not sure if a list item applies to your research, read the appropriate section before selecting a response.

Materials & experimental systems

n/a	Involved in the study	
☐	☒	Antibodies
☐	☒	Eukaryotic cell lines
☒	☐	Palaeontology and archaeology
☐	☒	Animals and other organisms
☒	☐	Human research participants
☒	☐	Clinical data
☒	☐	Dual use research of concern

Methods

n/a	Involved in the study	
☒	☐	ChIP-seq
☒	☐	Flow cytometry
☒	☐	MRI-based neuroimaging

Antibodies

Antibodies used	All antibodies were listed in Supplementary Table 3.
Validation	All antibodies were validated in previous studies and used in previous our study.<br><br>OCT4 (Mouse, Santa cruz #SC-5279 and SC-5279X): >1000 references are listed on the manufacturer's website.

OLIG2 (Rabbit, Merck Millipore #AB9610): >70 references are listed on the manufacturer's website.
NKX2.2 (Mouse, DSHB #74.5A5): >20 references are listed on the manufacturer's website.
A2B5 (Mouse, R&D Systems #MAB1416): 18 references are listed on the manufacturer's website.
PDGFRα (Rabbit, Santa cruz #SC-338): >100 references are listed on the manufacturer's website.
SOX10 (Goat, R&D Systems #AF2864): 18 references are listed on the manufacturer's website.
S100β (Mouse, Sigma-Aldrich #S2532): >100 references are listed on the manufacturer's website.
GFAP (Rabbit, Merck Millipore #AB5804): >100 references are listed on the manufacturer's website.
O4 (Mouse, R&D Systems #MAB1326): >100 references are listed on the manufacturer's website.
O4 (Mouse, clone 81, Merck Millipore #MAB345): > 100references are listed on the manufacturer's website.
MOG (Goat, Abcam #AB115597): > 20references are listed on the manufacturer's website.
MOG (Mouse, Merck Millipore #MAB5680): 4 references are listed on the manufacturer's website.
MBP (Rat, Abcam #AB7349): 98 references are listed on the manufacturer's website.
MBP (Rat, Merck Millipore #MAB386): >70 references are listed on the manufacturer's website.
PLP1 (Rabbit, Abcam #AB28486): 26 references are listed on the manufacturer's website.
TuJ1 (Mouse, BioLegend #801202): >150 references are listed on the manufacturer's website.
hNuclei (Mouse, clone235-1, Merck Millipore #MAB1281): >100 references are listed on the manufacturer's website.
hMitochondria (Mouse, clone 113-1, Merck Millipore #MAB1273): >40 references are listed on the manufacturer's website.
Neurofilament (Rabbit Sigma-Aldrich #N4142): >200 references are listed on the manufacturer's website.
GFP (Rabbit, Abcam #ab6556): >600 references are listed on the manufacturer's website.
GFP (Mouse, Abcam #ab1218): >200 references are listed on the manufacturer's website.

Eukaryotic cell lines

Policy information about cell lines

Cell line source(s)	H9-hESCs (WA-09) were obtained from WiCell Research Institute, SOX10::eGFP-hESCs were provided from Dr. Gabsang Lee (Johns Hopkins University), and OLIG2::eGFP-hESCs (R-Olig2) were provided from by Dr. Ying Liu (University of Texas). BJ (human foreskin fibroblasts) and HDFs (human dermal fibroblasts) were obtained from ATCC, hADSCs (human adipose derived stem cells) were obtained from Biostar Stemcell Research Institute, and hAFSCs (human amniotic fluid-derived stem cells) were obtained from our previous study.
Authentication	All human embryonic stem cell lines were authenticated by providers and also all fibroblasts were authenticated by providers or our laboratory members.
Mycoplasma contamination	All cell lines were negative for mycoplasma contamination, confirmed by their providers and our laboratory members.
Commonly misidentified lines (See ICLAC register)	No commonly misidentified lines are used in this study.

Animals and other organisms

Policy information about studies involving animals; ARRIVE guidelines recommended for reporting animal research

Laboratory animals	A total of 50 female mice (Eight-week-old C57BL/6, OrientBio) and 25 female mice (Four-week-old shiverer, The Jackson Laboratory).
Wild animals	None
Field-collected samples	None
Ethics oversight	All animal experiments including animal care and safety were performed with strict adherence to the guidelines of the Institutional Animal Care and Use Committee of Korea University (Seoul, South Korea).

Note that full information on the approval of the study protocol must also be provided in the manuscript.
